# Huntington’s disease LIG1 modifier variant increases ligase fidelity and suppresses somatic CAG repeat expansion

**DOI:** 10.1073/pnas.2518854123

**Published:** 2026-03-02

**Authors:** Eunhye Lee, Wonju Kim, David H. Beier, Yejin Lee, Marina Kovalenko, Faaiza Saif, Esaria Oliver, Bhairavi Srinageshwar, Ryan Murtha, Marissa A. Andrew, Andrew Jiang, Tammy Gillis, Brigitte Demelo, Jayla Ruliera, Diane Lucente, Seung Kwak, Ramee Lee, Ricardo Mouro Pinto, Marcy E. MacDonald, James F. Gusella, Patrick J. O’Brien, Vanessa C. Wheeler, Ihn Sik Seong

**Affiliations:** ^a^Molecular Neurogenetics Unit, Center for Genomic Medicine, Massachusetts General Hospital, Boston, MA 02114; ^b^Department of Neurology, Massachusetts General Hospital and Harvard Medical School, Boston, MA 02114; ^c^Department of Biological Chemistry, University of Michigan Medical School, Ann Arbor, MI 48109; ^d^CHDI Management Inc., Princeton, NJ 08540; ^e^Medical and Population Genetics Program, The Broad Institute of Massachusetts Institute of Technology and Harvard, Cambridge, MA 02142; ^f^Department of Genetics, Blavatnik Institute, Harvard Medical School, Boston, MA 02115

**Keywords:** Huntington’s disease, DNA ligase 1, repair fidelity, DNA damage, somatic repeat expansion

## Abstract

Huntington’s disease (HD) is a fatal neurodegenerative disorder caused by inheriting an expanded CAG repeat tract in the huntingtin gene (*HTT*) that further expands in somatic cells over an individual’s lifetime. Genome-wide association studies have provided critical insight into factors that modify the course of disease. These include DNA repair genes that alter the rate of somatic expansion and other genes that do not appear to directly influence this process. One modifier gene is DNA ligase 1 (*LIG1*), in which a variant specifying a lysine to asparagine substitution (K845N) is associated with a profound (7 to 8 y) delay in the onset of motor signs. Here, we have taken a multifaceted approach to gain insight into the protective nature of this variant in HD. We demonstrate using in vitro ligase assays and enzyme kinetics that K845N enhances discrimination toward mismatched substrates and increases repair fidelity. Consistent with increased ligation fidelity, K845N confers protection against oxidative stress in cell-based assays. Finally, we demonstrate that the mouse LIG1 K843N orthologue suppresses somatic CAG expansion in HD knock-in mice. Overall, our data provide evidence that altered LIG1 function due to the K845N substitution may contribute to HD clinical delay by slowing somatic expansion in the brain and protecting the genome globally against damage. Significantly, our results provide a mechanistic foundation for considering DNA ligase fidelity as a therapeutic target in HD and potentially in other trinucleotide repeat disorders.

## Significance

We analyzed a missense variant in DNA Ligase 1 (K845N) that is associated with a profound delay in the onset of Huntington’s disease (HD). We find that K845N enhances substrate discrimination toward mismatched substrates, thus increasing repair fidelity, conferring protection against oxidative stress and slows somatic expansion of the HD CAG repeat. Our observations provide insight into underlying mechanisms of disease modification and suggest avenues that can be harnessed for disease-modifying therapeutic intervention.

Huntington’s disease (HD) (MIM #143100) is a progressive, autosomal dominant neurodegenerative disorder characterized by motor dysfunction, cognitive decline, and behavioral disturbances (1). It is caused by inheriting an expanded CAG trinucleotide repeat (≥36 repeat units) within the first exon of the huntingtin gene (*HTT*) located on chromosome 4 (2). The expanded CAG repeat is unstable in somatic cells and progressively increases in length over the life of HD individuals, particularly in neurons in the brain (3–8). The length of the inherited CAG repeat is the most significant genetic factor influencing the age at motor onset (AAO) of HD, with longer repeat lengths associated, on average, with an earlier onset of motor symptoms (9–11). While repeat length accounts for a substantial portion (~60%) of the variation in AAO among individuals with HD, there remains considerable variability in AAO (± ~20 y) that is unexplained by inherited repeat length and which is heritable, indicating additional genetic factors that contribute to disease (10).

Identifying these modifying factors is essential to gain a comprehensive understanding of HD pathogenesis. Insights into these modifiers may improve predictions of the onset and progression of HD and inform the development of novel disease-modifying therapies. Genome-wide association studies (GWAS) have emerged as a powerful tool for systematically scanning the genomes of large cohorts of HD patients, enabling the identification of genetic variants associated with specific disease characteristics, such as AAO (12–17). Notably, these GWAS have identified genes involved in DNA repair pathways, in particular in mismatch repair (MMR), as modifiers of HD AAO and other clinical measures, and together with single cell-based and mechanistic studies, support a critical role for the somatic expansion of the CAG repeat in determining the timing of disease phenotypes (8, 18). Other modifier genes do not directly implicate somatic CAG expansion and may be involved in neuronal vulnerability/toxicity mechanisms (15, 17).

One of the genetic modifiers, associated with modification of multiple HD clinical landmarks, is DNA ligase 1 (*LIG1*) (chromosome 19q13.33), encoding LIG1. The most recent GWAS (17) defined two distinguishable *LIG1* modifier effects, named 19AM1 and 19AM3. The top single nucleotide variant (SNV), whose minor allele tags the 19AM3 modifier effect is rs145821638 (GRCh38 - Chr19:48117686-C-A), specifies a lysine to asparagine substitution in LIG1, at position 845 (K845N). This uncommon variant (minor allele frequency 0.18 in Europeans, ~0.14 across all gnomAD v4.1.0 populations) is associated with a 7 to 8-y delay in AAO, standing out as one of the strongest HD modifier effects detected to date. There are no other variants of comparable effect size and significance on the phased haplotype of the *LIG1* region from 19AM3 modifier chromosomes, arguing that K845N is the source of the modifier effect. The profound impact associated with this variant provides a strong rationale for understanding the mechanism of disease modification, and has potential to translate this knowledge into a therapeutic delay of disease onset.

LIG1 is an essential enzyme in mammalian development (19, 20) that catalyzes the formation of phosphodiester bonds to seal single-strand breaks in DNA. LIG1 plays a crucial role in completing DNA replication, where it joins Okazaki fragments on the lagging strand, and in DNA repair pathways, including base excision repair, nucleotide excision repair, and MMR (21, 22). High fidelity ligation by LIG1 is essential for maintaining the integrity of the genome by preventing the loss of genetic information and harmful mutations. Errors in ligation, particularly those involving mismatched bases, can lead to genome instability, which has been implicated in various diseases, including cancer and immunodeficiency (22).

LIG1 catalyzes the DNA ligation reaction through a three-step mechanism (23). In the first step, adenylylation, LIG1 reacts with Adenosine 5’ triphosphate (ATP) to covalently attach an adenosine monophosphate (AMP) moiety to a specific lysine residue in its active site (K568), forming a LIG1-AMP intermediate. The second step, adenylyl transfer, involves the transfer of AMP from LIG1 to the 5’ phosphate end of the DNA nick, resulting in an AMP-DNA intermediate. Finally, in the nick sealing step, the 3’ hydroxyl group at the DNA nick attacks the 5’ phosphodiester bond, displacing AMP and sealing the DNA backbone. The high fidelity of LIG1 relies on the ability of its active site residues to recognize and properly align the DNA ends, ensuring efficient and accurate ligation (24, 25). Some biallelic missense mutations in *LIG1* cause a recessive primary immune deficiency referred to as LIG1 Syndrome or IMD96 (MIM #619774) (26–28) associated with reduced LIG1 catalytic activity and altered DNA binding, highlighting potential for specific amino acid substitutions to alter LIG1 function. No such LIG1 Syndrome-associated mutations were identified in HD modifier GWAS cohorts, presumably due to their very low population frequencies (Table 1). However, homozygotes for the A allele specifying LIG1 K845N are observed in the population (gnomADv4.1.0) but this variant has not been reported to cause LIG1 Syndrome, hinting that any impact of the K845N change on LIG1 activity may be distinct from the effects causing LIG1 Syndrome. Indeed, such an impact may not be disease-causing per se but rather become evident only under particular circumstances, such as an intersection with the HD pathogenic mechanism.

**Table 1.** Minor allele frequency and relative ligase activity for LIG1 variants^*^

	WT	K845N	R768W^†^	R771W^‡^	R641L^‡^	R305Q^†^
dbSNP**^§^**	NA	rs145821638	rs371393033	rs121434561	rs34087182	rs1000596485
GRCh38 location-SNV	NA	19-48117686-C-A	19-48121253-G-A	19-48121244-G-A	19-48127920-C-A	19-48143543-C-T
Freq**^¶^**	(1)	1/700	1/26,000	1/38,000	1/17,000	1/440,000
Rel. *k*_cat_	(1)	0.38	0.46	0.055	0.10	0.31
Rel. *k_cat_*/*K_M_*	(1)	0.20	0.07	0.014	0.026	0.037

^*^Steady-state ligation assays for ∆232 LIG1 were performed with 50 mM NaMOPS, pH 7.5, 0.2 mM ATP, 1.2 mM MgCl_2_ (1 mM free Mg^2+^) at 37 °C. Steady-state parameters are given for formation of ligated product. Data for LIG1 WT and K845N are from this work.

^†^From ref. 29.

^‡^From ref. 30.

^§^The rsID may also include other less frequent SNVs that result in the same amino acid change.

^¶^Frequency of the minor allele in the overall population is from the gnomAD database v4.1.0 and is expressed as a fraction rounded to 2 significant figures (31).

The biochemical properties of the LIG1 K845N variant and its potential as a contributor to a mechanism that delays HD pathogenesis are unknown. In this study, we have taken a multifaceted approach to this question that includes in vitro biochemical, cell-based, and mouse studies. Our results demonstrate that LIG1 K845N reduces ligase activity toward mismatched substrates, with kinetic studies demonstrating increased ligation fidelity. In cell-based models of oxidative damage, we show protection by the K845N variant against cellular toxicity, consistent with a reduction in ligating promutagenic nicks. Finally, we demonstrate that the mouse ortholog of the human LIG1 K845N variant, LIG1 K843N, suppresses somatic CAG expansion in HD knock-in mice. Taken together, these comprehensive approaches provide insight into the role of the LIG1 K845N variant in maintaining genomic integrity, implicate mechanisms of action of this modifier variant and support further investigation to harness insights into K845N for modifying disease outcomes in HD.

## Results

### LIG1 K845N Shows Reduced Ligase Activity In Vitro That Is Sensitive to the Ligation Context, Particularly Promutagenic 3’ Mismatches.

**Fig. 1.**
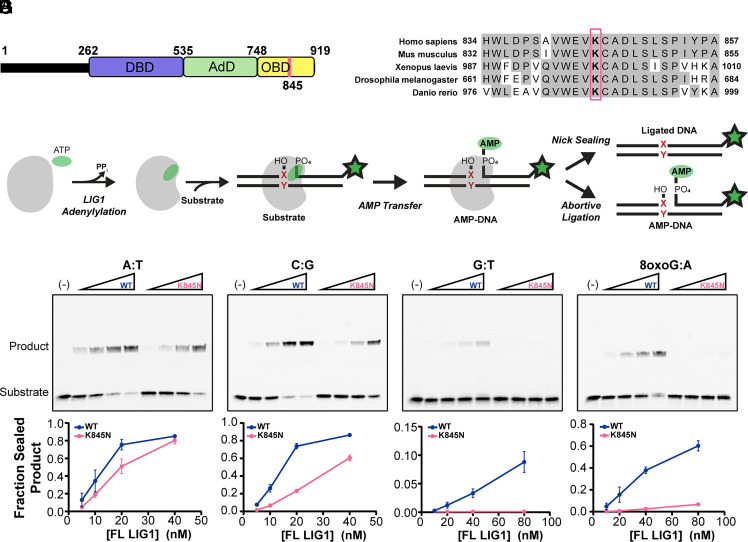
LIG1 K845N shows reduced ligase activity toward mismatched substrates. (*A*) Domain architecture of LIG1. The DNA binding domain (DBD), AdD, and OBD comprise the catalytic core. (*B*) Multiple sequence alignment of LIG1 orthologs by Clustal Omega. The position of K845 is indicated by the red box. (*C*) Illustration of LIG1 reaction. LIG1 is first adenylylated by ATP at K568, forming a LIG1-AMP intermediate. The second step, adenyl transfer, involves the transfer of AMP from LIG1 to the 5’ phosphate end of the DNA nick, resulting in a DNA-AMP intermediate. Finally, in the nick sealing step, the 3’ hydroxyl group at the DNA nick attacks the 5’ phosphodiester bond, displacing AMP and sealing the DNA backbone. LIG1 may also release premature AMP-DNA intermediates, known as abortive ligation. Our in vitro ligation reaction uses nicked substrate generated by annealing three oligonucleotides, one of which is labeled with FAM (green star), allowing substrate, aborted DNA-AMP, and ligated product to be distinguished. X:Y = canonical or mismatched base pair. (*D*–*G*) Representative gels and quantified fraction of ligation product showing the ligase activity of full-length LIG1 WT and K845N in the context of nucleotide pairs at the 3’ nick position. A:T (*D*), C:G (*E*), G:T (*F*), 8-oxoG:A (*G*) containing nicked 34mer DNA (300 nM) was incubated with increasing concentrations of LIG1 proteins for 5 min at 37 °C. Reactions were performed with 1 mM ATP, 10 mM MgCl_2_, 50 mM MOPS pH 7.5 and 150 mM NaCl. Reactants were analyzed on 15% TBE-urea polyacrylamide mini gels (note that in these gels substrate and AMP-DNA are not resolved). The line graphs show the quantification of the fraction of ligated product from three independent experiments (Mean ± SD).

The K845N variant of LIG1 substitutes a positively charged lysine with a polar asparagine within the conserved oligonucleotide binding-fold domain (OBD) that is essential for DNA binding and positioning (Fig. 1 *A* and *B*). K845 participates in interdomain interactions between the OBD and the adenylylation domain (AdD) (*SI Appendix*, Fig. S1). To investigate the impact of the K845N substitution on DNA ligase activity, we assessed the in vitro ligase activity of LIG1 with lysine at position 845 [LIG1 wild-type (WT)] and LIG1 with asparagine at position 845 (LIG1 K845N) using gel-based DNA ligation assays (Fig. 1*C*). These assays were performed using full-length LIG1 WT and K845N to evaluate ligation activity in the context of the intact enzyme. Full-length LIG1 WT and K845N proteins were purified in their adenylylated forms which allowed us to measure the concentration of active enzyme using single turnover ligation in the absence of ATP (i.e., active site titrations; *SI Appendix*, Fig. S2*C*). LIG1 WT and K845N showed similar active concentrations of 89% and 92%, demonstrating that the K845N substitution does not impact the purification of the adenylylated enzyme.

We compared ligase activity of full-length LIG1 K845N to WT in a standard ligase assay buffer containing a saturating amount of Mg^2+^ (~10 mM) and varying concentrations of enzyme (Fig. 1 *D*–*G*). We used 34mer canonical nicked DNA substrates with different nucleotides at the 3’ end of the nick (Fig. 1*C* and *SI Appendix*, Table S1). Ligase activity was quantified as the ratio of the intensity of the ligated DNA product relative to the summed intensities of the ligated DNA, aborted AMP-DNA, and unreacted substrate. Using a dA:dT (hereafter simplified to A:T) substrate, both LIG1 WT and K845N were catalytically active (Fig. 1*D*). In contrast, there was a modest, but significant decrease in ligase activity for K845N when a 3’ C:G substrate was tested (Fig. 1*E*). This pattern was also observed with T:A and G:C substrates, where complementary base pairing was reversed (*SI Appendix*, Fig. S3). From these data it is apparent that K845N is highly active as a DNA ligase and has context-dependent effects that differ from those of LIG1 WT.

LIG1-catalyzed nick sealing completes DNA replication and repair pathways, and therefore the ability to distinguish mismatches from correctly paired bases helps to maintain genomic integrity. We next tested the promutagenic contexts that would result from polymerase incorporation errors using all 12 possible mismatches at the 3’-OH side of the nicked DNA (Fig. 1*F* and *SI Appendix*, Figs. S4 and S5). Under our experimental conditions, the K845N variant showed no detectable ligation activity on any of the mismatched substrates, whereas LIG1 WT exhibited weak but detectable activity on the G:T, C:T, T:C, T:G, C:A, and A:C mismatches (Fig. 1*F* and *SI Appendix*, Fig. S4). For the remaining six base pair combinations, WT showed little or no ligase activity and K845N also showed no detectable ligase activity (*SI Appendix*, Fig. S5). Thus, while K845N displays reduced ligase activity for all substrates tested, this systematic screen demonstrates that the defect is disproportionately greater for mismatched substrates, resulting in near-complete suppression of ligation at promutagenic nicks.

LIG1 is known to be challenged in distinguishing an 8-oxoG:A mispair relative to other types of mismatches due to its stable Hoogsteen base pairing (25, 32–34) and yet this is a common promutagenic substrate formed under oxidative conditions. We therefore compared the activity of LIG1 K845N and WT in the 3’ 8-oxoG:A ligation context. As was observed for the nonoxidized mismatches, K845N showed reduced ligase activity with 8-oxoG:A compared to LIG1 WT (Fig. 1*G*). Reduced ligation activity toward replication errors such as mismatches or incorporation of 8-oxo-dGTP from the oxidized nucleotide pool could elevate the overall fidelity of genome maintenance and support a protective function of K845N.

### K845N Selectively Aborts Ligation of an 8-oxoG:A Mismatch to Enhance the Fidelity of Ligation.

**Fig. 2.**
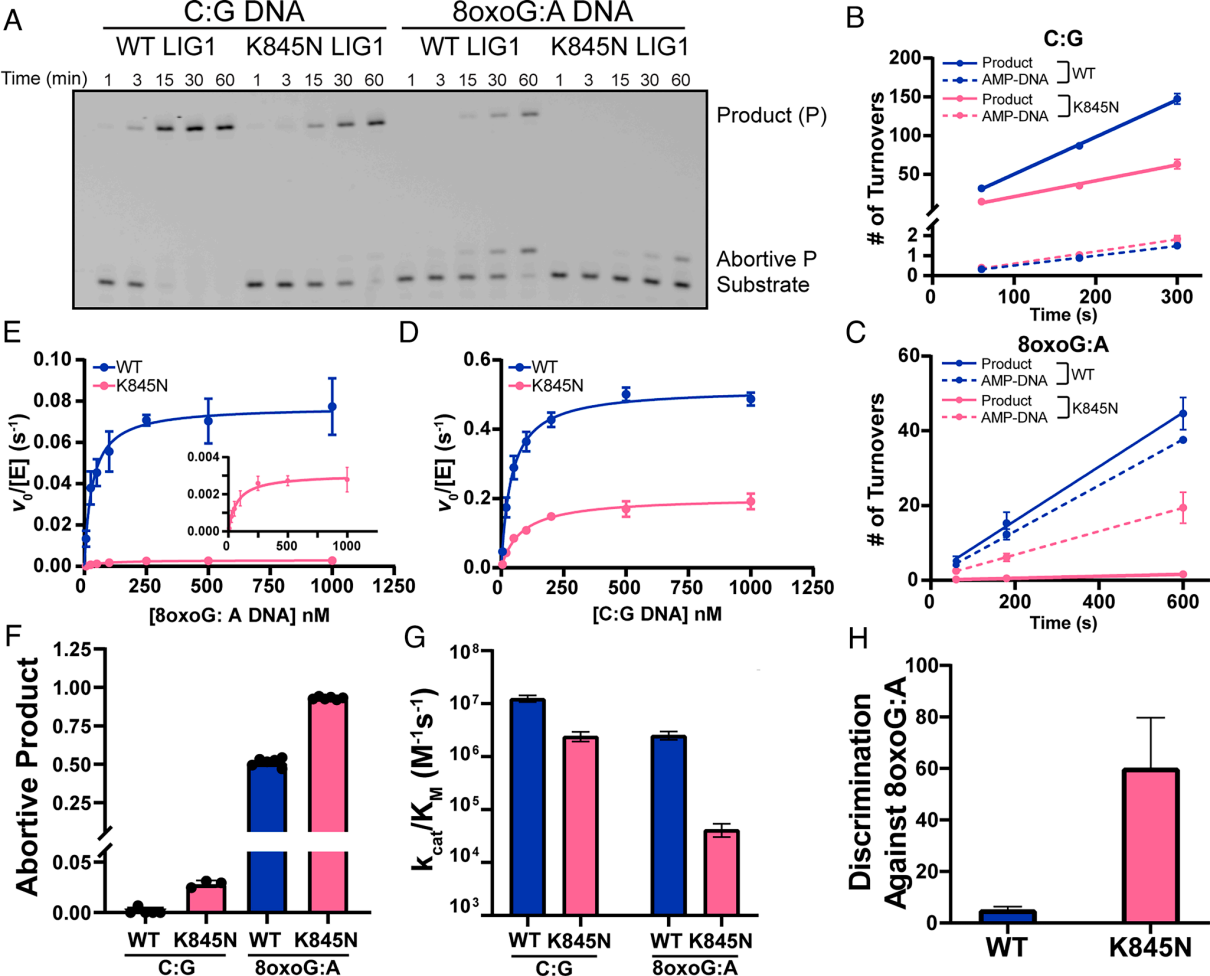
K845N is a higher fidelity, but lower activity LIG1 variant as determined by Michaelis–Menten kinetics. (*A*) Representative denaturing PAGE showing in vitro ligation reaction with WT or K845N ∆232 LIG1 (1 nM) with C:G or 8-oxoG:A containing nicked DNA (500 nM), resolving ligated product, aborted product, and substrate. Reactions were performed at 37 °C with 1 mM ATP, 2 mM MgCl_2_ (1.0 mM free Mg^2^). Time courses for ligation of 1,000 nM C:G (*B*) and 8-oxoG:A (*C*) 28mer substrate were plotted as number of turnovers with 1 and 5 nM LIG1 WT and 1.5 and 10 nM LIG1 K845N, respectively. Michaelis–Menten dependence for C:G (*D*) and 8-oxoG:A (*E*) 28mer nicked DNA was determined with 0.2 mM ATP and 1.0 mM free Mg^2+^. (*F*) The fraction of abortive ligation determined from initial rates of ligation. The catalytic efficiency for ligation (*G*) and the discrimination against 8-oxoG:A (*H*) were calculated from the kinetic parameters in panels *D* and *E*. Data reported are mean ± SD (N ≥ 3) and are summarized in *SI Appendix*, Table S2.

To elucidate how the K845N substitution alters the biochemical properties of LIG1, we performed a thorough kinetic characterization of ∆232 LIG1 under the same conditions that have previously been used to study other rare variants of LIG1 (26, 29, 30). This truncation removes the disordered and poorly conserved N terminus of LIG1 which contains the nuclear localization sequence (24, 35). Active site titrations were used to determine the concentration of active ∆232 LIG1 (*SI Appendix*, Fig. S6). Differential scanning fluorimetry showed that ∆232 LIG1 K845N has the same global stability as the WT protein (*SI Appendix*, Fig. S7). We next measured DNA binding using a previously described assay that employs a nicked DNA substrate in the absence of Mg^2+^ to prevent ligation (30). We found that ∆232 LIG1 K845N binds to a nicked DNA substrate with the same affinity as LIG1 WT (*SI Appendix*, Fig. S7*D*). We then investigated the catalytic activity of ∆232 LIG1 variants using steady-state kinetics. Representative time courses for DNA ligation are shown in Fig. 2*A* comparing LIG1 WT and K845N with an undamaged nicked DNA substrate (C:G) and a substrate containing an 8-oxoG:A at the 3’ end of the nick. Similar to what was observed for the full-length LIG1 at higher Mg^2+^ concentration with a different sequence context (Fig. 1 *E* and *G*), K845N exhibited reduced activity on the undamaged substrate and substantially less activity on the 8-oxoG:A substrate. Using high-resolution sequencing gels (denaturing polyacrylamide gel electrophoresis (PAGE)), the abortive AMP-DNA species that is particularly prevalent in reaction of the 8-oxoG:A substrate is resolved from unreacted substrate. We next measured the initial rates of ligation at saturating concentration of DNA (1,000 nM). Representative initial rates are shown for the canonical substrate in Fig. 2*B*. These data show that the reduced production of sealed DNA product in reactions catalyzed by K845N (solid lines) is accompanied by a detectable increase in the amount of AMP-DNA release (dashed lines; Fig. 2*B*). Analogous reactions performed with the 8-oxoG:A substrate show that AMP-DNA release by LIG1 WT is roughly on par with the amount of ligated DNA, whereas LIG1 K845N releases mostly AMP-DNA and fails to complete the ligation reaction (Fig. 2*C*).

We next measured initial rates of reaction for the canonical nicked DNA substrate for ∆232 LIG1 WT and K845N across a range of substrate concentrations and fit the Michaelis–Menten equation to the data (Fig. 2*D*). The derived steady-state rate constants are tabulated in *SI Appendix,* Table S2. The maximal rate constant (k_cat_) values were 0.52 ± 0.01 and 0.20 ± 0.01 s^−1^ for LIG1 WT and K845N, respectively (a 2.6-fold decrease; Fig. 2*D* and *SI Appendix,* Table S2). The K_Μ_ value is 1.9-fold higher for K845N (*SI Appendix,* Table S2), resulting in a 4.9-fold decrease in the catalytic efficiency (k_cat_/K_M_). These k_cat_/K_M_ values are plotted in Fig. 2*G*. Analogous experiments were performed to determine steady-state kinetic parameters for the 8-oxoG:A substrate (Fig. 2*E*). There is a stark difference between the ligase activity of K845N and WT with this substrate, with most of the difference in the k_cat_ values (25-fold decrease) and a modest increase in K_M_ (2.3-fold; *SI Appendix,* Table S2), which corresponds to a 58-fold defect in k_cat_/K_M_ (Fig. 2*G*). We quantified the fraction of abortive ligation for each enzyme with each substrate (Fig. 2*F*), revealing that K845N increases the amount of abortive ligation for both substrates, with a more pronounced reduction in ligated product for the 8-oxoG:A substrate than for the undamaged substrate. The fraction of abortive ligation increases by 1.8-fold, corresponding to a 7.0-fold reduction in the fraction ligated (F_lig_) of the 8-oxoG:A substrate relative to the WT enzyme (F_lig_^WT^/F_lig_^K845N^ = (1 − 0.51)/(1 − 0.93) = 0.49/0.07 = 7.0). Thus, the enhanced ability of LIG1 K845N to reject the 8-oxoG:A-containing nick is attributed to both a lowered rate of nick sealing and a higher rate of abortive release of AMP-DNA.

To quantify the degree to which the K845N substitution increases the fidelity of LIG1, we calculated the discrimination factor by which ∆232 LIG1 WT and K845N distinguish 8-oxoG:A from undamaged nicks (*SI Appendix*, Table S2). Consistent with previous reports, LIG1 WT struggles to discriminate an 8-oxoG:A pair from other Watson–Crick paired nicks (25, 32, 34). This is due to the ability of 8-oxoG:A to form a Hoogsteen basepair that is further stabilized by hydrogen bonding in the LIG1 active site (25). Our data show only a 5.1-fold discrimination for LIG1 WT in ligation of 8-oxoG:A versus a canonical paired nick (Fig. 2*H* and *SI Appendix,* Table S2). In contrast, K845N discriminates against 8-oxoG:A by a factor of 60-fold, which is 12-fold higher fidelity than the LIG1 WT enzyme (Fig. 2*H* and *SI Appendix,* Table S2). K845 is highly conserved among mammalian LIG1 homologs; however, these data suggest that the K845N substitution confers unique biochemical properties. With minimal deleterious effect on ligation of canonical substrates, K845N shows an enhanced fidelity in preventing ligation of an upstream mismatch.

### Cells Harboring the LIG1 K845N Variant Exhibit Increased Viability and Decreased Genomic DNA Mutation under Oxidative Stress.

**Fig. 3.**
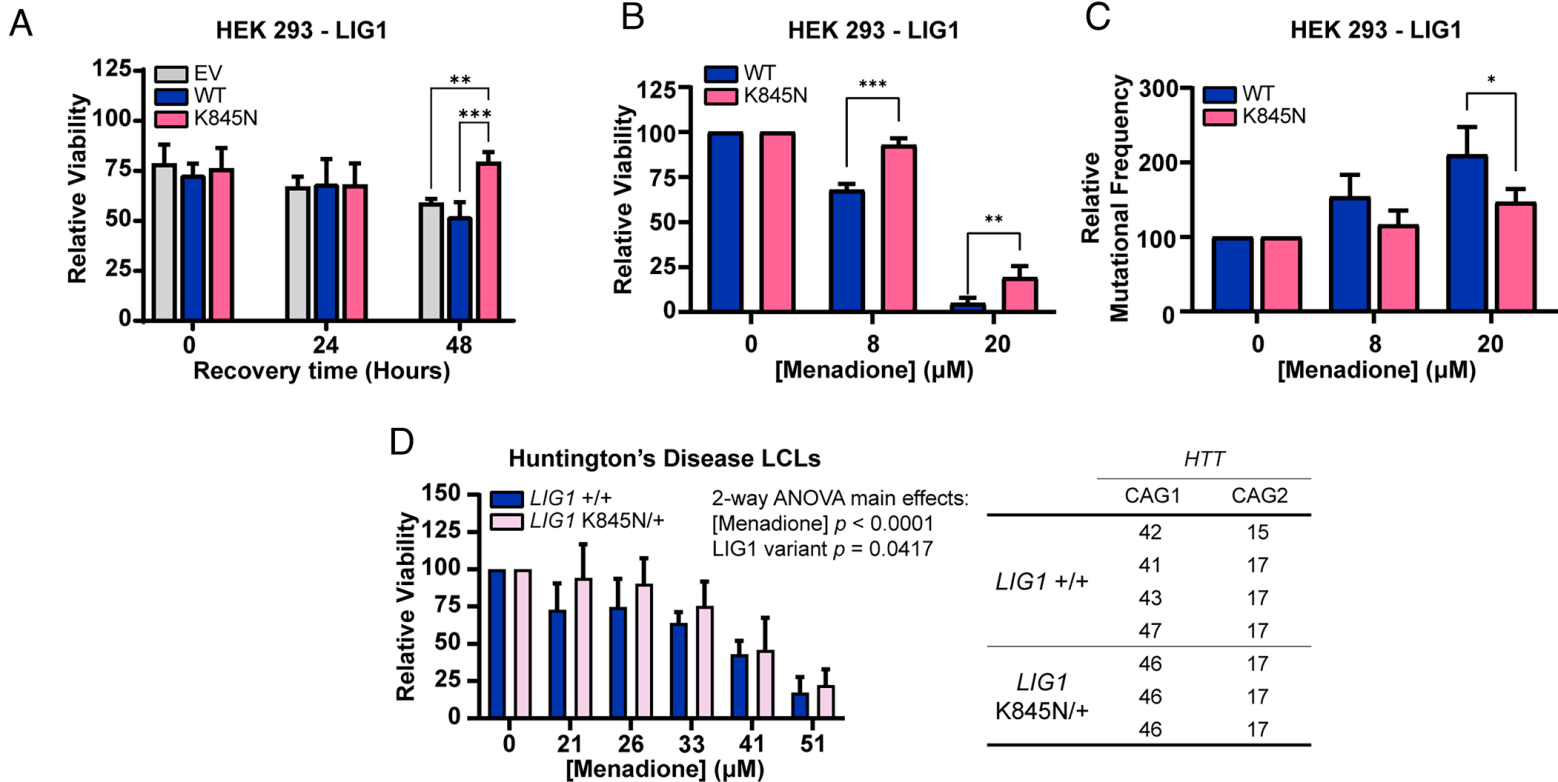
Cells harboring the LIG1 K845N variant exhibit increased viability and decreased DNA mutation under conditions that induce DNA damage. (*A*) HEK 293 cells [EV-transfected or expressing LIG1 WT or LIG1 K845N (K845N)] were treated with 8 µM menadione for 4 h. Cells were washed in fresh medium and further incubated for the indicated times. Cellular viability was measured by CTG. Bar graph shows mean of three biological replicates ± SD. Statistical significance was determined from two-way ANOVA with Tukey’s multiple comparison test (****P < 0.0001, **P < 0.01, *P < 0.05). (*B* and *C*) HEK 293 cells (WT or K845N) were treated with menadione for 4 h at the indicated concentrations and washed in fresh medium. After 48 h recovery, the cells were harvested. (*B*) A portion of the harvested cells was used to measure cell viability by CTG. Bar graph shows mean % viability relative to baseline (0% menadione) of two biological replicates ± SD. (*C*) DNA was extracted from the remaining cells and mutation frequencies were determined by duplex sequencing. Bar graph shows mean % MF relative to baseline (0% menadione) of two biological replicates ± SD. Statistical significance in *B* and *C* was determined from two-way ANOVA with Tukey’s multiple comparison test (*P < 0.05, **P < 0.01, ***P < 0.001). (*D*) LCLs derived from HD patients were treated with menadione for 4 h at the indicated concentrations. LCLs were either homozygous for the *LIG1* rs145821638 (GRCh38–Chr19:48117686) reference C-allele (*LIG1*+/+) or heterozygous for the 19AM3 modifier variant A-allele (*LIG1*K845N/+). Cells were washed in fresh medium and incubated for 24 h. Viability of each LCL was determined using CTG from the mean value of three biological replicates. Bar graph shows mean % viability relative to baseline (0% menadione) ± SD (n = 4 *LIG1*+/+ independent LCLs, n = 3 independent *LIG1*K845N/+ LCLs). Statistical significance indicated in figure was determined by two-way ANOVA with Tukey’s multiple comparison test. The table on the *Right* shows the *HTT* CAG repeat lengths for both alleles (CAG1 and CAG2) in HD patient derived LCLs carrying either the *LIG1* reference allele (*LIG1*+/+) or the heterozygous *LIG1* K845N variant (*LIG1* K845N/+).

Based on these biochemical studies, we hypothesized that the enhanced fidelity of LIG1 K845N leads to a reduction in cellular DNA mutation rate, providing a survival advantage under cellular stress conditions. Given previous data implicating oxidative stress as a potential contributor to HD (36), we tested whether K845N might protect against oxidative DNA damage-induced stress, first using human embryonic kidney (HEK) 293 cells stably overexpressing either full-length LIG1 WT or K845N at similar levels (*SI Appendix,* Fig. S9*A*). Cells were treated with 8 µM menadione that induces oxidative DNA damage, and viability was assessed immediately after treatment and after 24 or 48 h of recovery (Fig. 3*A*). Immediately after treatment, viabilities (~66%) were not different among HEK 293 cells transfected with empty vector (EV), and those expressing LIG1 WT or K845N. After 24 h of recovery, both LIG1 WT and K845N expressing cells showed slightly increased viabilities compared to EV-transfected cells. Interestingly, after 48 h of recovery, cells expressing K845N showed significantly higher viability than EV-transfected (*P* < 0.0001) or LIG1 WT-expressing cells (*P* < 0.0001). Furthermore, when assessed after 48 h of recovery at various concentrations of menadione, cells expressing K845N showed consistently higher viability across all treatment conditions (*SI Appendix,* Fig. S8*A*), implying that the K845N variant confers a recovery-dependent protective effect against oxidative stress. To test whether the recovery-dependent phenotype could reflect compensatory changes in the abundance of DNA ligases, we quantified LIG1, LIG3, and LIG4 protein levels in untreated and menadione-treated cells, with or without recovery but did not observe any significant differences, indicating that the observed phenotype is not attributable to altered DNA ligase expression (*SI Appendix*, Fig. S9 *A–D*). Further, LIG1 WT or K845N expressing cells showed no significant difference in cellular growth rate, indicating that intrinsic growth rates do not influence the differential cell viabilities (*SI Appendix*, Fig. S9*E*). To determine whether reduced ligase activity alone drives the K845N survival phenotype, we tested the catalytic-dead LIG1 mutant K568A (24, 37). Unlike K845N, K568A did not increase viability following menadione treatment and resembled WT cells (*SI Appendix*, Fig. S8 *C* and *D*), indicating that enhanced survival is not due to loss of catalytic activity but instead reflects a fidelity-dependent effect.

To directly assess genomic DNA mutation rates, we performed duplex sequencing on HEK 293 cells expressing either LIG1 WT or K845N. We reoptimized conditions for a scaled-up assay that would provide sufficient cells for duplex sequencing, establishing that under these conditions, cells expressing LIG1 K845N exhibited significantly higher viability when treated with 8 µM or 20 µM menadione followed by 48 h of recovery (Fig. 3*B*). We partitioned the cells for viability measurement and extracted DNA from the remaining cells. Duplex sequencing was performed to determine mutation frequency (MF). In both LIG1 WT- and LIG1 K845N-expressing cells MF increased in a menadione concentration-dependent manner (*SI Appendix,* Fig. S8*B*). While cells expressing LIG1 K845N exhibited a slightly higher baseline MF in the absence of treatment, they exhibited slightly lower MF in the presence of menadione. Relative to baseline (0 µM menadione), there were significant main effects (two-way ANOVA) of both menadione concentration (*P* = 0.0069) and LIG1 variant (*P* = 0.0377) on MF. Correcting for multiple comparisons revealed that the relative MF in K845N-expressing cells was significantly lower than in WT-expressing cells at 20 µM menadione (*P* = 0.0279), with a similar trend observed at 8 µM (Fig. 3*C*). These results suggest that the increased cellular viability conferred by K845N may be contributed by a decrease in mutation burden, consistent with the enhanced fidelity of K845N.

To extend these findings to HD patient-derived cells, we examined lymphoblastoid cell lines (LCLs) harboring only the *LIG1* rs145821638 (GRCh38 - Chr19:48117686) reference C-allele (*LIG1+/+*) or heterozygous HD LCLs harboring the 19AM3 modifier variant A-allele (*LIG1*K845N/+). We treated HD patient *LIG1*+/+ and *LIG1*K845N/+ LCLs with varying concentrations of menadione, and as with HEK 293 cells, viability was measured immediately, and after 24 and 48 h of recovery. While *LIG1*+/+ LCLs exhibited increased viability immediately following menadione treatment without recovery (*P* = 0.0402) (*SI Appendix*, Fig. S10*A*), *LIG1*K845N/+ LCLs showed significantly higher viability than *LIG1*+/+ LCLs after a 24-h recovery period postmenadione treatment (Fig. 3*D*). Relative to baseline (0 µM menadione), two-way ANOVA identified significant main effects of both menadione concentration (*P* < 0.0001) and K845N genotype (*P* = 0.0417) on cell viability, with no significant interaction between the two factors (*P* = 0.7225). This indicates that the effects of menadione treatment and genotype were additive, and that both genotypes exhibited similar viability responses across increasing menadione concentrations. No individual genotype comparison remained significant after multiple testing correction, though viability increased to the greatest extent in *LIG1*K845N/+ vs. *LIG1*+/+ LCLs treated with 21 µM menadione (*P* = 0.0589). After 48 h, differences were similar to those observed at 24 h but were less distinct (*SI Appendix*, Fig. S10*B*). Given the small number of available HD LCLs with the K845N variant—limited to heterozygous carriers of this rare variant, and the genetic background heterogeneity of the patient samples, the statistical power in the LCLs is limited. Nevertheless, consistent with findings in HEK 293 cells, where the protective effect of LIG1 K845N emerged after a recovery period, HD LCLs expressing the endogenous LIG1 K845N variant also exhibited a recovery-dependent protective phenotype. As in the HEK 293 overexpression system, we observed no difference in basal proliferation rate between *LIG1*+/+ and *LIG1*K845N/+ cells (*SI Appendix*, Fig. S10*C*) and no significant differences in LIG1, LIG3, and LIG4 protein levels in untreated and menadione-treated cells, with or without recovery. (*SI Appendix*, Fig. S11 *A*–*D*).

### The Mouse K845N Orthologous Variant Suppresses Somatic CAG Expansion in *Htt*^Q111^ Mice.

**Fig. 4.**
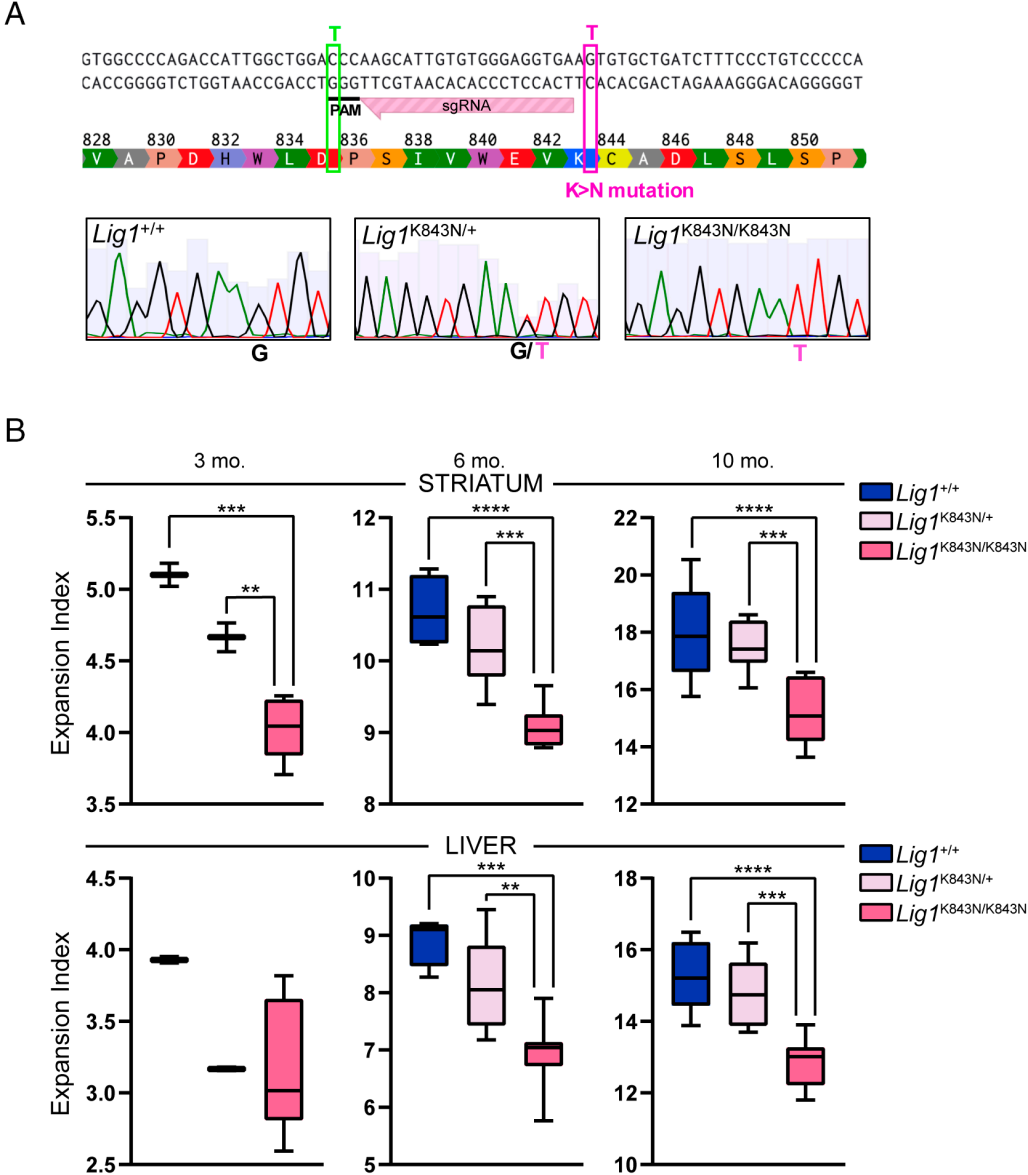
The orthologous K843N mutation suppresses somatic CAG expansion in mice. (*A*) CRISPR-Cas9-mediated generation of the *Lig1*^K843N^ knock-in allele. *Top*: The sequence is part of mouse *Lig1* exon 26 (canonical transcript ENSMUST00000177588.10) with amino acid translation showing lysine (K) at amino acid 843 (orthologous to human K845). The targeting sgRNA and PAM are shown. A 150 nt single-stranded oligonucleotide (not depicted in the Figure; refer to *Materials and Methods* for detail) was used for homology-directed repair to mutate G > A changing K843 to asparagine (N) (pink box). This also introduced a silent C > T change within the PAM (green box). *Bottom*: Sanger sequence from a *Lig1* WT mouse, and heterozygous and homozygous K843N knock-in mice. (*B*) Somatic CAG expansion indices (Min to Max box-whisker plots) from striatum and liver of *Htt*^Q111/+^ mice with different *Lig1* genotypes. 3 mo: *Lig1*^+/+^ N = 2, *Lig1*^K843N/+^ N = 2, *Lig1*^K843N/K843N^ N = 8. 6 mo: *Lig1*^+/+^ N = 4, *Lig1*^K843N/+^ N = 9, *Lig1*^K843N/K843N^ N = 7. 10 mo: Lig1+/+ N = 9, *Lig1*^K843N/+^ N = 9, *Lig1*^K843N/K843N^ N = 10. **P < 0.01; ***P < 0.001, ****P < 0.0001 (one-way ANOVA, comparing all genotypes for each age and tissue, with Tukey’s multiple comparison test).

The somatic expansion of the *HTT* CAG repeat drives the onset of clinical phenotypes in HD. Age-dependent somatic expansion is readily measured in *Htt*^Q111^ CAG knock-in mice where it occurs at a high rate in both the striatum and the liver (18, 38). We have previously shown that somatic expansion in both these tissues can be modified to different degrees by MMR gene and various other gene knockouts and can also be modified by missense mutation impacting protein function (18, 39–42). To test whether the *LIG1* missense variant associated with delayed HD impacts somatic CAG expansion, we used CRISPR-Cas9-mediated homology-directed repair to introduce the same mutation as the human modifier (19AM3, rs145821638 C->A) into the mouse *Lig1* gene, changing the LIG1 lysine residue at amino acid 843 to an asparagine (Fig. 4*A*). As highlighted in Fig. 1*B*, K845 is a conserved residue, indicating its functional importance across species, including the mouse. We derived a *Lig1*^K843N^ line, which we crossed with *Htt*^Q111^ mice. The *Lig1*^K843N^ variant did not impact embryonic development, with *Lig1*^+/+^, *Lig1*^K843N/+^, or *Lig1*^K843N/K843N^ born at expected Mendelian ratios (*SI Appendix*, Fig. S12), contrasting with *Lig1* null alleles where homozygous null embryos do not survive beyond E15.5/E16.5 (19, 20). Consistent with the viability of *Lig1*^K843N/K843N^ mice, the K843N change did not alter *Lig1* expression levels (*SI Appendix*, Fig. S13). We also did not observe any overt phenotype in *Lig1*^K843N/+^ or *Lig1*^K843N/K843N^ mice. The mice did not develop tumors, in contrast to mice harboring the LIG1 Syndrome-associated R771W point mutation that in patient cells (46BR1 cell line) reduces ligase activity 20-fold (43). *Htt*^Q111/+^ mice with *Lig1*^+/+^, *Lig1*^K843N/+^, and *Lig1*^K843N/K843N^ genotypes were aged for analyses of somatic expansion. The two most unstable tissues, striatum, and liver (Fig. 4*B* and *SI Appendix*, Fig. S15) revealed significantly reduced somatic expansion in *Lig1*^K843N/K843N^ striata compared to *Lig1*^+/+^ striata at 3, 6, and 10 mo of age (3 mo *P* < 0.001, 6 and 10 mo *P* < 0.0001) and significantly reduced somatic expansion in *Lig1*^K843N/K843N^ liver at 6 and 10 mo of age (6 mo *P* < 0.001, 10 mo *P* < 0.0001). Expansion was also significantly reduced in *Lig1*^K843N/K843N^ cortex at 3 mo (*P* < 0.001) and 6 mo of age (*P* < 0.0001) and in the hippocampus at 6 mo of age (*P* < 0.001) (*SI Appendix*, Figs. S14 and S15). There were no significant differences in the inherited repeat lengths in the cohorts of mice with different *Lig1* genotypes (*SI Appendix*, Fig. S16). Further, expansion was not impacted by the presence of a silent protospacer adjacent motif (PAM) mutation cointroduced with K843N, as determined in an independent line of mice harboring the PAM mutation in the absence of the K843N change (*Materials and Methods* and *SI Appendix*, Fig. S17). Of the tissues analyzed, LIG1K843N appeared to have the most robust impact on expansion in the striatum. Although we observed *Lig1*^K843N^ allele dose-dependent trends, expansion was not significantly impacted in heterozygous *Lig1*^K843N/+^ mice relative to *Lig1*^+/+^ mice. The impact of this variant appears to be moderate, with one variant allele reducing striatal expansion by 3 to 5% and two mutant alleles reducing expansion by ~15 to 16% in 6 to 10-mo mice. Overall, these data support reduced somatic expansion as a consequence of a LIG1 functional alteration elicited by the K843N substitution.

## Discussion

The minor allele of rs145821638, coding for the *LIG1* K845N variant, was identified in GWAS as the top SNV capturing a modifier effect associated with delayed HD clinical phenotypes (13, 17). The impact of this SNV is substantial, delaying motor onset by 7 to 8 y. This rare variant, present in approximately 1 in 700 individuals (across all populations in gnomAD v.4.1.0), substitutes a conserved lysine within the OBD with a polar asparagine predicted to disrupt hydrogen bonding interactions at the domain interface between the OBD and the AdD. In this study, we evaluated the hypothesis that the HD modifier effect reflects mechanisms directly impacted by this *LIG1* K845N missense change by studying its biochemical, cellular, and molecular consequences.

Biallelic loss of function *LIG1* missense mutations associated with LIG1 Syndrome exhibit decreases in the maximal rate constant and in the catalytic efficiency for DNA ligation (Table 1). In comparison, our biochemical analyses of full-length and ∆232 truncated forms of LIG1 reveal the impact of K845N to be distinct from *LIG1* alleles associated with immunodeficiency syndrome (Table 1). We find that K845N maintains near-normal ligation activity on undamaged nicked DNA substrates but exhibits reduced activity toward mismatched substrates. This enhanced substrate discrimination, including against 8-oxoG:A base pairs, highlights K845N as a higher-fidelity variant of LIG1. Kinetic studies demonstrate a 59-fold reduction in catalytic efficiency (k_cat_/K_M_) on 8-oxoG:A substrates compared to WT, accompanied by an increase in abortive ligation events. Notably, the K845N variant exhibits 60-fold discrimination between mismatched and correctly paired substrates, far exceeding the fivefold discrimination for WT.

Importantly, much of this discrimination manifests as increased abortive ligation of the improper nick which will prevent binding by another ligase and ensure additional time for other repair processes to take place. Thus, the enhanced ligation fidelity of K845N is predicted to manifest in a heterozygous background (i.e., even if cells express both LIG1 WT and K845N). This is relevant in HD patients where profound clinical modification is seen in individuals heterozygous for this *LIG1* variant. It should also be noted that AMP-DNA intermediates are toxic to certain primary neurons in the absence of aprataxin, a hydrolase that cleaves AMP-DNA linkages to regenerate the 5’ phosphate (44). Loss of function mutations in aprataxin cause Ataxia with Oculomotor Apraxia type 1 (45, 46). Given the prevalence of the K845N variant it is unlikely that abortive ligation overwhelms the ability of aprataxin to repair AMP-DNA sites in the brain. Nevertheless, how AMP–DNA intermediates are handled across different cellular contexts, and how this balance influences survival during recovery from oxidative stress, will require future investigation. For example, delayed ligation could influence DNA damage signaling during the recovery phase. LIG1 has been reported to interact with checkpoint-associated complexes such as hRad17–RFC, which promotes loading of the 9-1-1 clamp at sites of DNA damage (47). Slower or more selective ligation at damaged nicks could therefore extend engagement of checkpoint or stress-response pathways, providing additional time for repair.

**Fig. 5.**
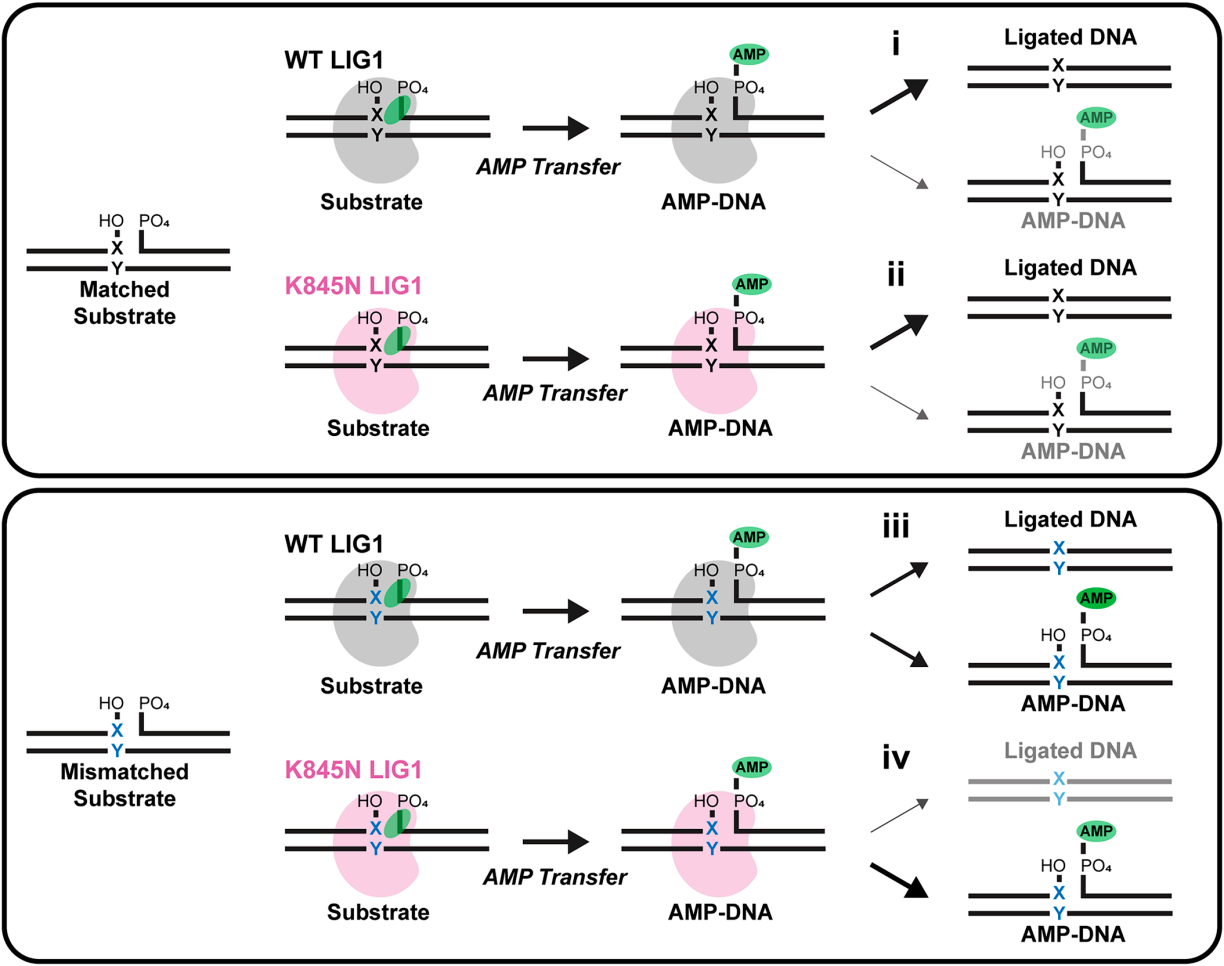
Schematic illustrating increased fidelity of ligation due to the K845N substitution. *Top* panel: Ligation of canonical base-paired substrate (black X:Y pair). (i) LIG1 WT produces mostly fully ligated DNA product (thick arrow and black depiction), with a small amount of aborted AMP-DNA (thin arrow and gray depiction). (ii) LIG1 K845N does not have a large impact on the proportions of ligated DNA and AMP-DNA. *Bottom* panel: Ligation of mismatched substrate (blue X:Y). (iii) LIG1 WT produces both fully ligated DNA product and aborted AMP-DNA (intermediate arrows). (iv) In contrast, LIG1 K845N produces less ligated product (thin arrow and gray depiction) and releases more aborted AMP-DNA (thick arrow and black depiction).

The K845N substitution is predicted to alter the interface between the AdD and the OBD which is expected to stabilize the closed conformation of LIG1 when it encircles the DNA substrate (*SI Appendix*, Fig. S1). We hypothesize that disruption of the interactions between K845 and the backbone amides of L750 and V753 will lead to increased flexibility and shift the bound conformation toward a more open conformation that allows for dissociation from the AMP-DNA intermediate. Opening of the OB-fold domain (OBD) in the LIG1 DNA complex has been observed in molecular dynamics simulations (48). This concept is illustrated in Fig. 5 (*Bottom*) whereby the increased dissociation from the AMP-DNA intermediate for a mismatched substrate provides the basis for enhanced ligase fidelity. Under the conditions tested, K845N retains a sufficiently stabilized closed complex that dissociation from the matched substrate is minimal and only a modest reduction in catalytic efficiency is observed (Fig. 5, *Top*).

Functionally, this fidelity enhancement translated into a measurable benefit in cellular assays. HEK 293 cells expressing LIG1 K845N exhibited significantly greater viability than LIG1 WT expressing cells following treatment with the oxidative damaging drug menadione. Notably, this protective effect was most pronounced after an extended recovery period, suggesting that the benefits conferred by K845N may arise gradually, potentially through enhanced DNA repair processes that require time to manifest. Consistent with this, K845N-expressing cells also demonstrated a reduced MF. Supporting the relevance of this observation in a disease context, HD patient-derived LCLs heterozygous for the K845N variant showed a modest increase in viability following menadione-induced stress after recovery. Overall, our findings provide evidence that K845N can enhance genome maintenance presumably through increased ligation fidelity.

Importantly, *Htt*^Q111^ knock-in mice carrying the orthologous *Lig1* missense variant (encoding LIG1 K843N) show a significant reduction in CAG repeat expansion in multiple tissues including the striatum and cortex that are particularly vulnerable in HD. The impact on expansion was modest; expansion was significantly suppressed in homozygous *Lig1*^K843N/K843N^ mice and trended in the same direction in heterozygous *Lig1*^K843N/+^ mice. Thus, on the face of it, the degree of expansion suppression in the mice may seem insufficient to account for the strong disease-delaying effect in patients heterozygous for this variant. However, as clinical onset is driven by somatic expansion that occurs over decades of life (8), the cumulative impact of small modifying effects may be substantial. Indeed, a profound reduction of somatic expansion, such as that elicited by MMR gene knockouts (18), would be unexpected over a 10-mo period in mice. Our study has only examined the impact of the LIG1 variant on the somatic expansion of ~100 CAG repeats, and an interesting possibility is that its effect may depend on CAG length, for example having different relative impacts on the recently described slow and rapid phases of somatic expansion (8). Interestingly, though *LIG1* variants modify clinical HD phenotypes, a recent GWAS of HD CAG repeat expansion in the blood did not identify significant signals at this locus. This suggests that, like several other loci that impact on either clinical phenotypes or blood CAG expansion but not the other, the *LIG1* locus can involve cell-type specific effects (17). Analyses of single cell gene expression data in human and mouse indicate that DNA LIG1, as well as DNA ligases 3 and 4, seem to be expressed at higher levels in stable striatal cell types, i.e., not medium spiny neurons (*SI Appendix*, Fig. S18). The impact of the *Lig1*^K843N^ variant on transcriptional dysregulation and on pathological markers (e.g., mutant *HTT* aggregation) in individual cell types will be a subject of future research. Overall, our data in mouse provide evidence that the mechanism underlying the 19AM3 disease-delaying effect may be contributed, at least in part, by an impact of the LIG1 K845N missense variant slowing the rate of somatic expansion in the brain.

It is of interest that neither knockout of *Lig1* nor the presence of the orthologous *Lig*1 R771W variant that reduces ligase activity 20-fold had any impact on the somatic expansion of CAG/CTG repeats in mouse models of HD and myotonic dystrophy type 1 respectively, though LIG1 levels modified CAG instability in an in vitro assay and the R771W variant promoted maternal germline contractions (18, 43, 49, 50). Thus, our data provide evidence for a role of LIG1 in the instability of CAG repeats in somatic tissues. These observations suggest that K845N may have unique biochemical properties that suppress somatic CAG expansion. While the precise mechanisms of somatic repeat expansion are not fully delineated, human and mouse genetic studies as well as biochemical data support a model in which expansions are driven by the recognition and processing of extruded CAG/CTG loops/structures by the MMR machinery (17, 18, 42, 51, 52). In this model (depicted in ref. 17) CAG/CTG loop-outs are substrates for MutSβ (MSH2-MSH3) binding followed by recruitment of MutLγ (MLH1-MLH3) endonuclease, DNA incision on the non-loop-containing strand, DNA excision, gap-filling, and ligation. Other factors, notably FAN1, counteract this expansion-prone mechanism by nicking DNA adjacent to the loop out. The presumed ligase concluding this process is LIG1 due to its involvement in MMR (53). We also have no direct evidence that *Lig3* modifies expansion, while knockout of *Lig4* promotes expansion in *Htt*^Q111^ mice (17). It is currently unclear how the LIG1 K845N variant might suppress expansion, and whether it acts at the final ligation step of a MMR-directed mechanism, or alternatively, acts at an earlier step in the process. One hypothesis is that the elevated fidelity that this variant confers might mitigate expansion by rejecting certain substrates that support further expansion (e.g., mismatched bases resulting from oxidative damage, previously implicated in CAG expansion (54, 55). Repeat-containing structures or flaps may themselves mimic mismatched DNA, and an alternative hypothesis is that failure to ligate certain substrates might directly promote pathway(s) that suppress expansion. e.g., LIG1 K845N may limit religation of FAN1-induced nicks adjacent to a repeat slip-out. Further mechanistic and biochemical studies to understand how K845N suppresses expansion will be a subject of future study.

In summary, our data demonstrate that the K845N variant of LIG1 confers enhanced substrate discrimination and increased repair fidelity, suppresses somatic CAG expansion and is associated with increased cell survival and decreased genome DNA MF independent of repeat expansion. As somatic expansion is a key disease driver, the suppression of somatic expansion by LIG1 K845N provides a plausible mechanistic underpinning for the HD-delaying 19AM3 modifier effect. Our findings also argue that the 19AM3 modifier effect may reflect, in part, increased cell survival due to genome-wide protection against DNA damage under conditions of genotoxic stress. These two potential (and nonmutually exclusive) mechanisms merit further investigation. Taken together, our results provide a mechanistic foundation for considering DNA ligase fidelity as a therapeutic target in HD and potentially in other trinucleotide repeat disorders.

## Materials and Methods

### Protein Expression and Purification.

Full-length and Δ232 LIG1 WT and K845N proteins were expressed and purified using established protocols to generate catalytically active enzyme for biochemical assays (see the details in *SI Appendix*, *Materials and Methods*). Protein purity and integrity were assessed by SDS-PAGE followed by Coomassie Blue staining. In addition, protein quality was further verified by western blotting using three independent LIG1 antibodies recognizing distinct epitopes, confirming comparable integrity of WT and K845N proteins (*SI Appendix*, Fig. S2 *A* and *B*).

### Preparation of DNA Substrates.

The DNA oligonucleotides were synthesized by Integrated DNA Technologies (IDT) and gel purified. Additional experimental details are provided in *SI Appendix*. The sequences of oligonucleotide substrates are listed in *SI Appendix*, Table S1.

### Active Site Titration.

To determine the active concentration of LIG1 in solution, active site titration assays were utilized (35). Briefly, full-length (12.5 nM to 400 nM) and Δ232 (20 nM to 300 nM) LIG1 variants were combined with 100 nM annealed assay substrate in the absence of ATP to prevent multiple turnover. This reaction was conducted in standard reaction buffer containing 50 mM MOPS pH 7.5, 1 mM DTT, 0.5 mg/mL BSA, 10 mM MgCl_2_, with the ionic strength adjusted to 150 mM using NaCl. After incubation at 37 °C for 1 h, reactions were quenched with standard loading buffer (90% formamide, 50 mM EDTA, 0.01% bromophenol blue, 0.01% xylene cyanol) and were heated to 95 °C for 5 min. The sealed product was separated from the substrate by denaturing PAGE on a 15% polyacrylamide/8 M urea gel. Active enzyme concentration was determined by segmental linear regression analysis in GraphPad Prism software.

### Gel-Based Ligation Assays for Full-Length LIG1.

The ligation assays for full-length LIG1 were performed in 150 mM NaCl, 50 mM MOPS (pH 7.5), 1.0 mM DTT, 0.5 mM ATP, 10 mM MgCl_2_, 300 nM nicked DNA substrate. Purified LIG1 (final 5 to 40 nM) was added to ligation buffer and the mixture was incubated for 5 min at 37 °C. Ligation reactions were quenched by cooling to 4 °C followed by addition of loading buffer (90% formamide, 50 mM EDTA), heated at 95 °C for 5 min. The samples were separated on 15% denaturing urea acrylamide mini gels and the gel was scanned with iBright imaging system (Thermo Fisher Scientific). Fluorescence intensity was analyzed using image Lab software (Bio-Rad). The fraction of observed ligation product was determined by dividing band intensity of product by the total band intensity.

### Steady-State Kinetics with ∆232 LIG1.

Steady-state kinetics experiments were performed with ∆232 LIG1 at 37 °C in standard reaction buffer with 1.2 mM MgCl_2_ (1 mM free Mg^2+^), 0.2 mM ATP, 50 mM MOPS pH 7.5, ionic strength adjusted to 150 mM using NaCl, and varying LIG1 and DNA concentrations. Each reaction time point was quenched in standard loading buffer and heated to 95 °C before gel loading. Sealed and abortive products were separated from substrate by denaturing PAGE on 15% polyacrylamide/8 M urea sequencing gels. Fluorescent bands were detected using an Amersham Typhoon 5 imager and quantified with ImageQuant TL software (Cytiva).

Initial rates were determined by fitting the fraction product (<0.20) to straight lines in GraphPad Prism, calculating reaction velocity from the slope. The fraction of abortive ligation is defined by Eq. **1**. The initial rates of sealed product formation were fit with the Michaelis–Menten equation (Eq. **2**) to determine *k*_cat_ and K_M_ values, as well as the catalytic efficiency (*k*_cat_/K_M_). The discrimination between 8-oxoG:A-containing substrate and undamaged (C:G) substrate is given by Eq. **3**. In some cases, the time courses are expressed as the number of turnovers, which is calculated by dividing the concentration of ligated product by the concentration of enzyme.

[1] $$\mathrm{Abortive}\;\mathrm{Product}=\frac{\left[\mathrm{Intermediate}\right]}{\left[\mathrm{Intermediate}\right]+[\mathrm{Sealed}\;\mathrm{Product}]},$$

[2] $$\frac{v_{0}}{\left[Enz\right]}=\frac{k_{\mathrm{cat}}[S]}{K_{M}+[S]},$$

[3] $$\mathrm{Discrimination}=\frac{\mathrm{Catalytic}\;{\mathrm{Efficiency}}_{C:G}}{\mathrm{Catalytic}\;{\mathrm{Efficiency}}_{8\mathrm{oxoG}:A}}.$$

### HD Patient and Control LCLs.

We used a panel of LCLs from seven previously collected and genotyped HD individuals participating in HD research. Of these, three were heterozygous for the 19AM3 modifier variant rs145821638 (GRCh38 - Chr19:48117686-C-A), all with *HTT* CAG repeat lengths 46/17, and four lacked the minor (A) allele, with *HTT* CAG repeat lengths 42/15, 41/17, 43/17, and 47/17. We also analyzed LCLs from two unaffected individuals lacking the 19AM3 minor allele (*HTT* CAG repeat lengths 17/15 and 20/19). LCLs were obtained from the MGB IRB-approved CHGR Neurodegenerative Repository. Genotyping of the normal and expanded CAG repeats was performed using a standardized PCR assay (56). *LIG1* SNV rs145821638 (GRCh38 - Chr19:48117686-C-A) genotype was determined using a TaqMan SNP genotyping assay from Life Technologies. The reaction contained 1× TaqMan Genotyping Master mix (Life Technologies, part # 4371355) 1× of custom TaqMan SNP Genotyping assay mix [Assay mix comes at 40× is diluted to 20× in TE buffer pH8.0 (Life Technologies part #AM9849)] and 10 ng of DNA in total reaction volume of 12.5 μL. The reaction was run on the Roche LightCycler 480 II with the following conditions: 95 °C for 10 min, followed by 35 cycles of 92 °C for 15 s, 60 °C for 1 min. Data were collected at the end of each amplification cycle and known homozygous WT and heterozygous controls were run with every plate to assign alleles.

### Cell Viability Assay and Duplex Sequencing.

To generate HEK 293 cells stably overexpressing either full-length LIG1 WT, K845N or K568A, HEK 293 cells were transfected in 24-well plates with pcDNA3.1 vectors encoding N-terminal FLAG-tagged full-length LIG1 WT, FLAG-tagged full-length LIG1 K845N, FLAG-tagged full-length LIG1 K568A or an EV using Lipofectamine 3000 (Life Technologies). The K845N and K568A construct was generated by site-directed mutagenesis (New England Biolabs) of the pcDNA3.1-LIG1 WT plasmid. After transfection, cells were subjected to G418 (Geneticin, Gibco) selection to enrich transfected cells. Following antibiotic selection, heterogeneous pooled populations were used for all experiments without further single-clone isolation. Independently maintained cultures of these heterogeneous populations, which were passaged and handled separately, were treated as biological replicates.

For cell viability assays, cells were seeded into 96-well plates at a density of 2 × 10^4^ cells/well in DMEM medium, and incubated overnight at 37 °C. The cells were treated with multiple dilutions of menadione (MP biomedicals) for 4 h, washed in fresh medium and incubated for indicated durations at 37 °C with 5% CO_2_. The number of viable cells was measured using the CellTiter-Glo (CTG) cell viability assay (Promega) according to the manufacturer’s instructions. Three technical replicate measurements were performed for each biological replicate. The mean of the technical replicate measurements was used for statistical analyses across the biological replicate samples. The data were expressed as the percentage of the amount of ATP measured relative to untreated controls.

For duplex sequencing, HEK 293 cells were seeded into six-well plates at a density of 8 × 10^5^ cells/well in DMEM medium, and incubated overnight at 37 °C. The cells were treated with 8 and 20 μM of menadione (MP biomedicals) for 4 h, washed in fresh medium and incubated for 48 h under normal conditions. After incubation, cells were harvested. A portion of the harvested cells was taken and cell viability was measured using CTG as above. The remaining cells were pelleted by centrifuge and stored at −80 °C until analysis. The cell pellets were sent to inotiv (https://www.inotiv.com) for genomic DNA extraction, library preparation, duplex sequencing, and MF analysis. Additional experimental details are provided in *SI Appendix*.

LCLs were grown in a humidified incubator in RPMI 1640 medium (Sigma-Aldrich) supplemented with 10% fetal bovine serum and 1% Penicillin-Streptomycin-Glutamine at 37 °C and 5% CO_2_. LCLs were seeded into 24-well plates at a density of 5 × 10^5^ cells/well in RPMI 1640 medium and treated with multiple dilution of menadione (The highest concentration was 100 μM and serial dilution of 4/5 were made) for 4 h, after which the medium was replaced with fresh medium for indicated recovery periods. The number of viable cells was determined using CTG as above. Three technical replicate measurements were performed for each biological replicate (different LCLs). The mean of the technical replicate measurements was used for statistical analyses across the biological replicate samples. The viability was expressed as the percentage of the amount of ATP measured relative to untreated controls.

### Generation of *Lig1*^K843N^ Knock-in Mice.

This study was carried out in accordance with the recommendations in the Guide for the Care and Use of Laboratory Animals of the NIH under an approved protocol of the Massachusetts General Hospital Institutional Animal Care and Use Committees (IACUCs) (MGH: protocol 2009N000216). All animal procedures were carried out to minimize pain and discomfort, under the approved IACUC protocol. Animal husbandry was performed under controlled temperature, humidity, and light/dark cycles.

CRISPR/Cas9-mediated homology-directed repair was carried out essentially as reported (57) (Genome Modification Facility of Harvard University). Candidate single guide RNAs (sgRNAs) were chosen using the Broad Institute design tool (58, 59), four sgRNAs screened in embryos using Sanger sequencing to test guide efficiency, from which one was picked based on having the highest estimated editing efficiency. This gRNA (5’>3’ 5’ TCACCTCCCACACAATGCTT; GRCm39 chr7:13,043,112-13,043,131 [- strand]) was coinjected into C57BL/6 J zygotes with Cas9 protein (Alt-R® S.p. Cas9 Nuclease V3, IDT) and the following 150 nt single-stranded oligonucleotide donor (ssODN) template (Genewiz) complementary to the guide sequence.

5’ TCTGCTCACTGCAGGCCCTGGTATTGCCTACCCCACGCCCCTATGTGAGGATTGATGGGGCAGTGGCCCCAGACCATTGGCTGGA***T***CCAAGCATTGTGTGGGAGGTGAA**T**TGTGCTGATCTTTCCCT

The ssODN harbored the coding G > T mutation (bold font) specifying asparagine at position 843 in the mouse LIG1 protein (in *Lig1* exon 26, canonical transcript ENSMUST00000177588.10) and a silent G > T mutation (italic font) within the S.p. Cas9 PAM sequence (converting the GGG PAM on the minus strand to GGA) to prevent endonuclease cleavage of the recombined sequence. Injected zygotes were implanted into pseudopregnant females and pups screened at weaning by Sanger sequencing for the presence of the targeted allele. We established germline transmission from a pup that exhibited ~100% of targeted sequence and further verified the presence of the targeted mutations (K843N and silent PAM) and absence of other sequence variants using MiSeq (primers F: 5’-CAGGCCCTGGTATTGCCTAC; R: 5’- CCATCACCTCTGCCTTCCTT). The line was subsequently maintained in crosses with C57BL/6J and sperm frozen at the Jackson laboratories. We also serendipitously obtained a line harboring only the silent PAM mutation, which we maintained in the same way and used as an additional control line. Mice were genotyped using custom designed TaqMan SNP genotyping assays from Life Technologies, specific for either the K843N variant or the PAM sequence variant. Reactions contained 1× TaqMan Genotyping Master mix (Life Technologies, part # 4371355) 1× of custom TaqMan SNP Genotyping assay mix (Assay mix comes at 40× is diluted to 20× in TE buffer pH 8.0 (Life Technologies part #AM9849) and 10 ng of DNA in total reaction volume of 12.5 µL. The reaction was run on the Roche LightCycler 480 II with the following conditions: 95 °C for 10 min, followed by 35 cycles of 92 °C for 15 s, 60 °C for 1 min, and data collected at the end of each amplification cycle. Known WT, heterozygous, and homozygous mutant controls were run with every plate to assign alleles.

### Mouse Crosses with *Htt*^Q111^ and Measurement of Somatic CAG Expansion.

*Htt*^Q111^ mice on the C57BL/6J genetic background (60) were crossed with *Lig1^K843N^* mice to generate *Htt*^Q111/+^ mice with *Lig1*^+/+^, *Lig1*^K843N/+^, and *Lig1*^K843N/K843N^ genotypes. Separate crosses were set up between *Htt*^Q111/+^ mice and the “PAM only” mutant line to generate a small cohort of *Htt*^Q111/+^ PAM/PAM control mice. *Lig1* alleles were genotyped as above and the *Htt*^Q111^ allele genotyped according to Kovalenko et al. (61). Genomic DNA from mice at 3, 6, or 10 mo of age was isolated from tissues using the DNeasy DNA blood and tissue kit (Qiagen) and somatic CAG instability analysis was performed using a human-specific PCR assay that amplifies the *HTT* CAG repeat from the knock-in allele (41). The forward primer was fluorescently labeled with 6-FAM (Thermo Fisher Scientific) and products were resolved using the ABI 3730xl DNA analyzer (Thermo Fisher Scientific) with GeneScan 500 LIZ as internal size standard (Thermo Fisher Scientific). GeneMapper v5 (Thermo Fisher Scientific) was used to generate CAG repeat size distribution traces. CAG expansion indices were calculated from GeneMapper peak height data as previously described, using a 5% relative peak height threshold cut-off (62). Expansion indices in all tissues were determined relative to the modal allele in the liver trace from the respective mouse (representing the modal repeat length in a population of liver cells that is very stable over time and distinguished as mice age from the unstable hepatocyte population (38).

### Assaying *Lig1* Expression in Mice.

Total RNA was extracted from 25 to 30 mg of mouse liver using a hybrid Trizol-Qiagen method (modified from https://imageslab.fiu.edu/wp-content/uploads/2020/02/RNA-extraction_Trizol_RNeasy_hybrid.pdf). The tissue was homogenized in Trizol reagent followed by phase separation according to Trizol manufacturer’s protocol. The top (aqueous) phase was mixed with two parts of RLT Plus buffer and applied to genomic DNA Eliminator spin columns (both buffer and columns were from the RNeasy Plus Mini kit, Qiagen, 74134). All the following steps were performed according to the RNeasy Plus Mini kit protocol. Quality control of resulting RNA was done with Agilent TapeStation 3000. The cDNA was synthesized with the Superscript III kit (Invitrogen, 18080-051) using oligo(dT) primer, according to the manufacturer’s instructions. The relative expression level of *Lig1* was quantified by PCR, using 2 µL of undiluted cDNA, 18 µL Taqman FastAdvanced Master Mix (Invitrogen, 4444556), and 1 µL of a ready-to-use Taqman assay from Invitrogen (*Lig1*: Mm00495331_m1 mapping to exons 21 to 22 and Mm01199942_m1 mapping to exons 3 to 4). PCR conditions were as follows: UNG incubation: 50 °C (2 min), initial denaturation and polymerase activation: 95 °C (20 s), 40 cycles of 95 °C (3 s), 60 °C (30 s), final extension 72 °C (10 min). Relative expression values (DC_t_) of *Lig1* for each mouse were calculated using geometric mean of the C_t_ values of three housekeeping genes (*Ppia*, Mm02342430_g1; *Actb*, Mm00607939_s1; *Gusb*, Mm00446953_m1). Expression fold change (2^−DDCt^) of *Lig1* for each mouse was calculated relative to the geomean of relative expression values (DC_t_) of the control group (*Lig1^+/+^*) mice.

### Statistical Analyses.

Statistical analyses were performed using GraphPad Prism v10. For comparisons of menadione-induced effects between LIG1 WT and LIG1 K845N-expressing cells, two-way ANOVA followed by Tukey’s multiple comparison test were conducted. This analysis was applied to both cell viability assays and MF measurements obtained from duplex sequencing. Proliferation rates of HEK 293 EV, LIG1 WT, and LIG1 K845N were analyzed by linear regression, whereas proliferation rates of LCLs were assessed using a mixed effect model to account for interindividual variability. For quantification of LIG1, LIG3, and LIG4 expression, one-way ANOVA with Tukey’s multiple comparison test was used for HEK 293 EV, LIG1 WT, and LIG1 K845N, while comparisons between the two LCL groups were evaluated using the unpaired *t* test with Welch’s correction. Comparisons of somatic expansion and *Lig1* expression levels in mouse tissues were carried out using one-way ANOVA with Tukey’s multiple comparison test. Mendelian ratios of pups with different *Lig1* genotypes were assessed with chi-squared tests. Significance was represented using a symbol-based threshold system: ****P < 0.0001, **P < 0.001, **P < 0.01, and *P < 0.05.

## Supplementary Material

Appendix 01 (PDF)

## Data Availability

Duplex sequencing data have been deposited in the NCBI Sequence Read Archive (SRA) under BioProject accession PRJNA1425166 (63). All other data supporting the finding of this study are included in the article and/or *SI Appendix*.
