# Supplementary material for: Huntington’s disease LIG1 modifier variant increases ligase fidelity and suppresses somatic CAG repeat expansion
Source: Proc Natl Acad Sci U S A. 2026 Mar 2;123(10):e2518854123. doi: 10.1073/pnas.2518854123 (PMC12974472; doi:10.1073/pnas.2518854123)
Supplement: Supplementary file 1 — Appendix 01 (PDF) [file pnas.2518854123.sapp.pdf]

Supplementary Methods and Figures

Huntington's disease LIG1 modifier variant increases ligase fidelity and suppresses somatic CAG repeat expansion

Eunhye Lee[1,2#], Wonju Kim[1,2#], David H. Beier[3#], Yejin Lee[1,2], Marina Kovalenko[1], Faaiza Saif[1], Esaria Oliver[1], Bhairavi Srinageshwar[1,2], Ryan Murtha[1], Marissa A. Andrew[1], Andrew Jiang[1,2], Tammy Gillis[1], Brigitte Demelo[1], Jayla Ruliera[1], Diane Lucente[1,2], Seung Kwak[5], Ramee Lee[5], Ricardo Mouro Pinto[1,2,4], Marcy E. MacDonald[1,2,4], James F. Gusella[1,2,4,6], Patrick J. O'Brien[3*], Vanessa C. Wheeler[1,2,4*], Ihn Sik Seong[1,2*]

1. Molecular Neurogenetics Unit, Center for Genomic Medicine, Massachusetts General Hospital, Boston, MA, 02114
2. Department of Neurology, Massachusetts General Hospital and Harvard Medical School, Boston, MA, 02114
3. Department of Biological Chemistry, University of Michigan Medical School, Ann Arbor, MI, 48109
4. Medical and Population Genetics Program, the Broad Institute of Massachusetts Institute of Technology and Harvard, Cambridge, MA, 02142
5. CHDI Management Inc., Princeton, NJ, 08540
6. Department of Genetics, Blavatnik Institute, Harvard Medical School, Boston, MA, 02115

[#]These authors contributed equally to this work.

*Correspondence: pjobrien@umich.edu (P.J.O.), vwheeler@mgh.harvard.edu (V.C.W.), iseong@mgh.harvard.edu (I.S.S.)

Materials and Methods

Antibodies

The following antibodies were used: anti-LIG1 (Abcam ab227133, Proteintech 18051-1-AP, Invitrogen MA5-42920), anti-alpha tubulin (Cell Signaling Technology, 3873S), anti-LIG3 (Cell Signaling Technology 43640S), anti-LIG4 (Cell Signaling Technology 14649S) and anti-alpha tubulin (Cell Signaling Technology 3873S).

Protein expression and purification

N-terminal FLAG-tagged full-length LIG1 WT and K845N were cloned into the pET28a vector and expressed in *Escherichia coli* BL21(DE3) at 18°C in the presence of 0.5 mM Isopropyl β-D-1-thiogalactopyranoside (IPTG) for 18 hours. Cells were harvested in 500 mM NaCl, 50 mM Tris pH 8.0, 1 mM EDTA, 5 % Glycerol in presence of cOmplete™, Mini, EDTA-free Protease Inhibitor Cocktail (Roche). The cells were lysed, and the lysates were cleared by centrifugation at 15,000 rpm for 40 minutes. The supernatant was incubated with M2-affinity resins (Sigma-Aldrich) for 4 hours and the proteins were eluted with 0.4 mg/mL FLAG peptide. The eluted samples were dialyzed into 150 mM NaCl, 50 mM Tris pH 8.0, 5% Glycerol and concentrated using Amicon Ultra centrifugal filter devices (Millipore). Protein purity and integrity were assessed by SDS-PAGE followed by Coomassie Blue staining. In addition, protein quality was further verified by western blotting using three independent LIG1 antibodies recognizing distinct epitopes, confirming comparable integrity of WT and K845N proteins.

Δ232 LIG1 was expressed from a pET19 plasmid vector (1), and the K845N variant was created using site-directed mutagenesis. Sequences were confirmed by full plasmid sequencing. LIG1 variants were expressed via auto-induction in TB + 0.2x trace metals, 0.2% lactose, 0.05% glucose, 0.4% glycerol and 5 µg/mL carbenicillin (2). After 4 hours of shaking at 37 °C, cells were pelleted and diluted with an equal volume of lysis buffer (50 mM Tris-Cl pH 7.5, 10% glycerol, 300

mM NaCl, 5 mM β-mercaptoethanol) and protease inhibitors were added (0.5 mM PMSF, 0.5 μg/mL leupeptin, 0.7 μg/mL pepstatin A, 0.01% IGEPAL). Cells were then lysed with a cell homogenizer, and collected via centrifugation (19,000 rpm, 30 minutes at 4 °C with an SS-34 rotor). PEI precipitation removed excess nucleic acids (0.1% poly(ethyleneimine) *v/v* cell supernatant while stirring at 4 °C). After the soluble fraction was centrifuged again, LIG1 was purified via standard low-pressure His-Trap column. Briefly, a 5 mL Ni-NTA column was first washed with a high-salt buffer (20 mM HEPES pH 7.5, 20 mM imidazole, 500 mM NaCl), followed by a low-salt buffer (20 mM HEPES pH 7.5, 50 mM imidazole, 100 mM NaCl). The soluble fraction was then loaded onto the column, followed by a low-salt buffer wash. Finally, proteins were eluted into fractions via a gradient between low- and high-imidazole buffers (20 mM HEPES pH 7.5, 50-300 mM imidazole, 100 mM NaCl). LIG1 adenylation and His-tag cleavage took place overnight at 4 °C with 1 mM ATP, 11 mM $MgCl_2$ 0.5 mM EDTA and purified precision protease enzyme. Next, LIG1 was further purified via FPLC HiTrap Q column using low salt (20 mM HEPES pH 7.5, 1 mM EDTA, 5 mM β-mercaptoethanol) wash buffer and high salt (20 mM HEPES pH 7.5, 1 mM EDTA, 2 M NaCl, 5 mM β-mercaptoethanol) elution buffer using a buffer gradient. LIG1 was then purified again via HiTrap Blue column using the same buffers and method as the HiTrap Q column. Finally, purified Δ232 LIG1 variants were buffer exchanged into storage buffer (25 mM Tris-Cl pH 7.5, 150 mM NaCl, 0.1 mM EDTA, 1 mM DTT) and purity was confirmed via SDS-PAGE.

Preparation of DNA substrates

The DNA oligonucleotides were synthesized by Integrated DNA Technologies (IDT) and gel purified. Concentrations were determined by the absorbance using the predicted A_{260} values. The nicked DNA substrates were generated by annealing the oligos in a 1:1.5:2 ratio (10 μM PO4 to 15 μM template to 20 μM OH) in buffer comprised of 10 mM NaMES (pH 6.5) and 50 mM NaCl.

The mixture was incubated at 95 °C for 5 minutes and then gradually cooling the solution to 4 °C, decreasing the temperature by 1°C every 5 seconds. Annealed substrates were stored at 4°C.

Duplex sequencing

Genomic DNA from the cell pellets was isolated from harvested cells and assessed for integrity by agarose gel electrophoresis and for purity by NanoDrop spectrophotometry. DNA concentration was determined using Qubit assays, and samples not meeting quality thresholds were further purified using SPRI bead cleanup. Up to 650 ng of genomic DNA was used as input for library preparation when available. DNA libraries were prepared using the TwinStrand Duplex Sequencing™ Mutagenesis Panel (Human-50), v2.0 kit (TwinStrand Bioscience, Seattle, WA, USA) according to the manufacturer's protocol and inotiv RTP standard operating procedures. Genomic DNA was enzymatically fragmented and ligated to double-stranded adapters containing unique molecular identifiers (UMIs) on both strands, enabling duplex consensus error correction. Libraries were indexed, amplified by limited PCR, purified, and assessed for size and quality using an Agilent Bioanalyzer prior to pooling. Libraries were pooled at equimolar concentrations and sequenced on a single lane of an Illumina NovaSeq X Plus 25B flow cell using paired-end 150-bp reads, targeting ~542 million paired-end reads per sample (~1–1.25 billion duplex base pairs per sample). Sequencing data were processed using TwinStrand DuplexSeq™ software (v4.5.0) within the DNAnexus platform to generate duplex consensus reads. Mutation frequencies were calculated using the minimum mutation counting method, considering only subclonal single-nucleotide variants (variant allele frequency <1%), and defined as the number of mutant duplex bases divided by total duplex bases per sample. Statistical analyses were performed across the biological replicate samples (replicate sequencing for each sample was not performed).

Expression analysis of LIG1, LIG3 and LIG4 under menadione treatment

Expression of LIG1, LIG3 and LIG4 under menadione treatment was examined in HEK 293 stable overexpression lines and lymphoblastoid cell lines. Analysis in HEK 293 cells (EV, LIG1 WT and LIG1 K845N) were performed under the same treatment and cell seeding conditions used for duplex sequencing. HEK 293 cells were treated with 20 μM menadione for 4 h. Cells were harvested either immediately (no recovery) or following 24 h or 48 h recovery in fresh medium. Two biological replicate cultures for each condition were used.

LCLs (*LIG1* +/+ and *LIG1* K845N/+) were seeded in 6 well plates at 2 x 10^6 cells per well and treated with 21 μM menadione for 4 h, followed by collection under the same three recovery conditions (0, 24 or 48 h). Cell lysates were prepared in RIPA buffer and analyzed by SDS-PAGE and western blotting using antibodies against LIG1, LIG3 and LIG4. α-tubulin served as a loading control. Band intensities were quantified by normalizing each target protein to α-tubulin.

Proliferation assay

Proliferation rates of HEK 293 EV, LIG1 WT, LIG1 K845N and LIG1 K568A cells were measured by seeding 1.5 x 10^4 cells per well in 12 well plates and culturing for 7 days. Cell numbers were determined daily using Trypan blue exclusion and a Countess automated cell counter (Thermo Fisher Scientific). Doubling times were calculated using data from days 3 to 7, corresponding to the linear growth phase, and statistical significance among the three conditions was assessed by linear regression analysis.

For HD LCLs, cells were seeded at 2.0 x 10^5 cells per well in 6 well plates and cultured for 6 days, with daily cell counting using trypan blue and the Countess automated cell counter. Doubling times were calculated using data from days 1 to 4. Differences between the two groups (*LIG1* +/+ and *LIG1* K845N/+) were evaluated using a mixed effects model. Two biological replicate cultures were used.

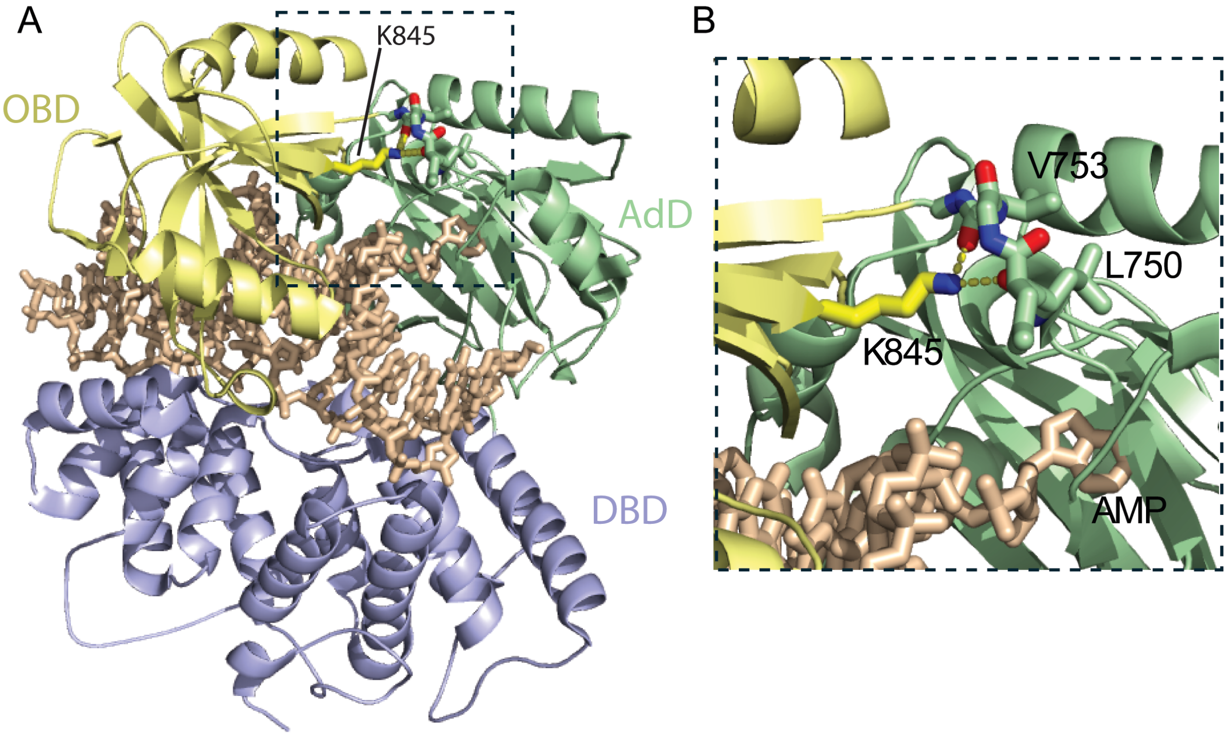

Figure S1. Interdomain contacts made by K845 in the complex of LIG1 bound to DNA. (A) LIG1 WT in complex with adenylylated DNA rendered from PDB 6p0c (3). The DNA binding domain (DBD; purple), adenylylation domain (AdD; green), and OB-fold domain (OBD; yellow) encircle the AMP-DNA intermediate (brown). The dashed box centers on K845 from the OBD and is enlarged below. (B) The epsilon amino group of K845 makes bidentate hydrogen bonds with the backbone carbonyls of L750 and V753 of the AdD (2.4 and 2.7 Å respectively, yellow dashed lines). Thus, K845 forms a lynchpin at the interface between the OBD and AdD in the DNA-bound complex of LIG1. By removing these hydrogen bonding interactions, it is expected that the K845N substitution will destabilize the closed complex of LIG1 bound to DNA.

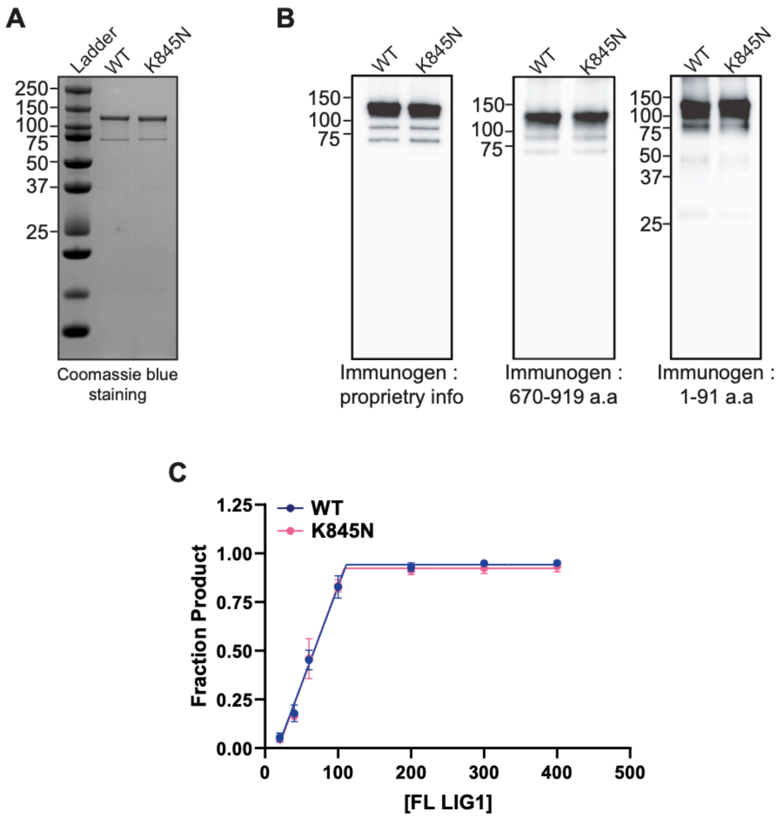

Figure S2. Purification and active concentration of full-length LIG1 WT and K845N. (A) Full-length LIG1 WT and K845N proteins were analyzed by 10% SDS-PAGE stained with Coomassie blue. Both proteins migrated slightly above 100 kDa, consistent with full-length LIG1, and showed an indistinguishable pattern of minor contaminants. (B) Full-length LIG1 WT and K845N were analyzed by western blot using three antibodies recognizing distinct regions of LIG1: antibody 1 (immunogen undisclosed), antibody 2 (a.a. 670–919), and antibody 3 (a.a. 1–91). All antibodies detected the full-length species above 100 kDa and one or more fragments at ~75-kDa fragment. Antibody 3 detected very low concentrations of presumably N-terminal truncated fragments of LIG1. The almost identical patterns observed for the two proteins indicate that WT and K845N exhibit comparable protein quality. (C) Active site titration assays were performed to measure the concentration of active LIG1 using 100 nM of A:T (34mer) nicked DNA substrate in the absence of ATP. Reaction products were resolved by 15% TBE-Urea polyacrylamide gels. The line graphs show the quantification of the fraction of ligated product from three independent experiments (mean ± SD). WT and K845N were determined to be 89 ± 6% and 92 ± 6% active, respectively.

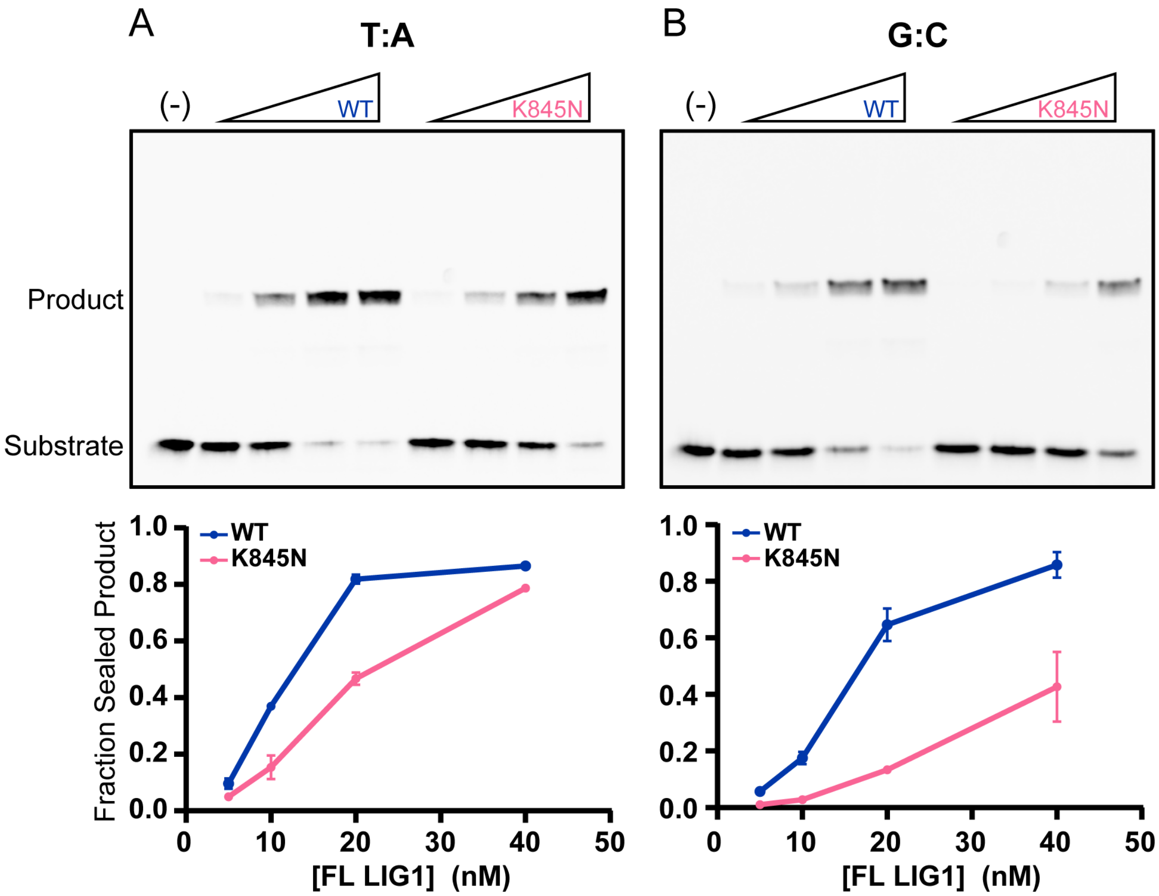

Figure S3. Ligation of canonical substrate by LIG1 WT and K845N. Representative gels showing the ligase activity of LIG1 WT and K845N. T:A (A) or G:C (B) containing nicked DNA (34mer; 300 nM) was incubated with increasing amounts of the proteins for 5 min at 37 °C. Reactants were analyzed in 15% TBE-Urea polyacrylamide gels. Reactions were performed with 1 mM ATP, 10 mM $MgCl_2$, 50 mM MOPS pH 7.5 and 150 mM NaCl. The line graphs show the quantification of the fraction of ligated product from three independent experiments (mean ± SD). Note that in panel (A), the error bars for the WT group are smaller than the symbol size and may not be visible.

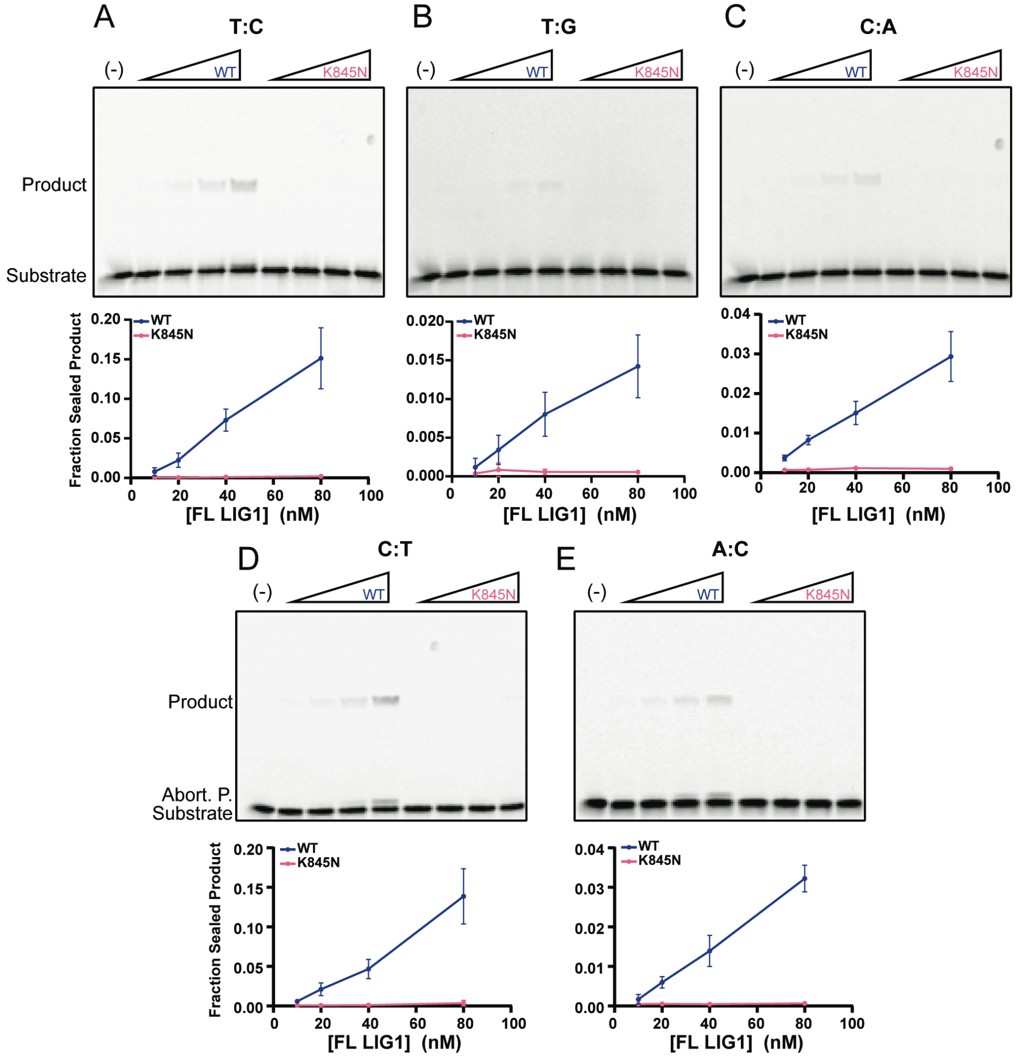

Figure S4. Ligation of mismatched substrate by LIG1 WT and K845N. Representative gels showing the ligation of mismatched DNA substrates by LIG1 WT and K845N. T:C (A), T:G (B), C:A (C), C:T (D), and A:C (E) containing nicked DNA (34mer) was incubated with increasing amounts of enzyme for 5 min at 37 °C. Reactions were performed with 1 mM ATP, 10 mM $MgCl_2$, 50 mM MOPS pH 7.5 and 150 mM NaCl. Reactants were analyzed by 15% TBE-Urea polyacrylamide gels. The line graphs show the quantification of the fraction of ligated product from three independent experiments (mean ± SD).

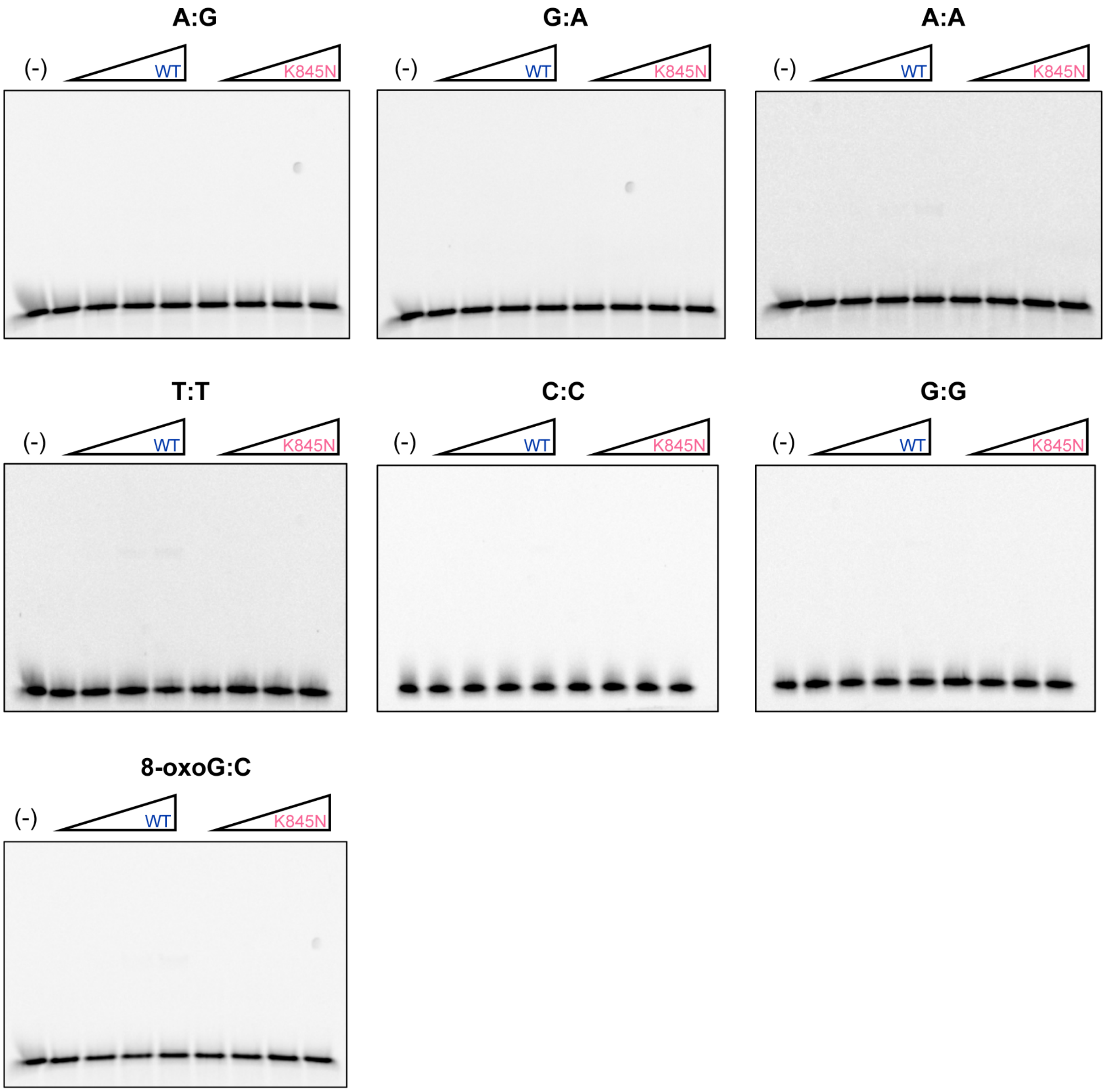

Figure S5. Ligase assays with additional mismatched substrates. Representative gels showing the absence of significant ligated product for several different 3' X:Y 34mer contexts. DNA (34mer; 300 nM) was incubated with 10-80 nM enzyme for 5 min at 37 °C. There was no detectable product formed by either LIG1 WT or K845N, and therefore no quantitation was performed.

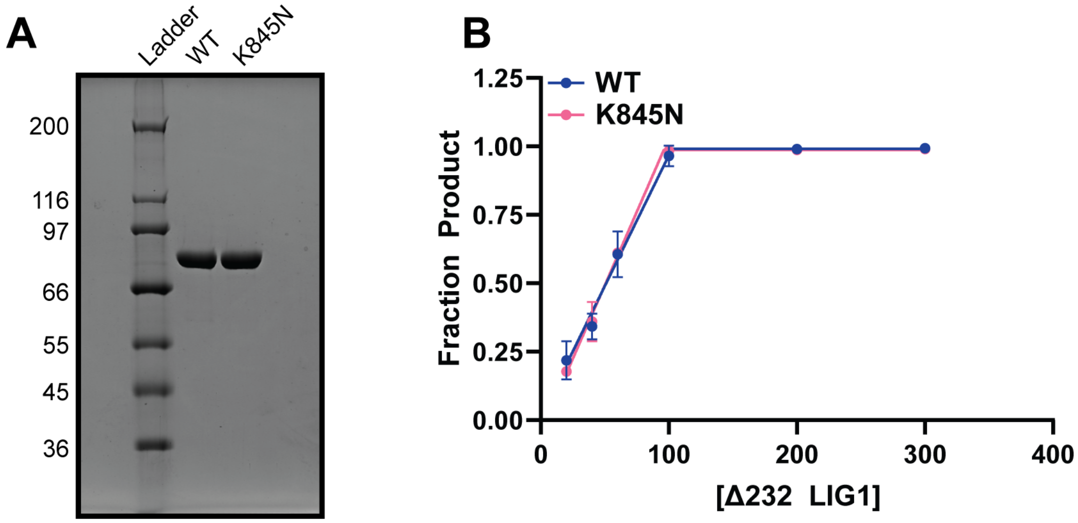

Figure S6. Purification and active concentration of WT and K845N Δ232 LIG1. (A) Purified LIG1 WT and K845N proteins (1 µg per lane) were analyzed with 10% SDS-PAGE (Δ232 LIG1 is 76.5 kDa). (B) An active site titration assay was conducted in the absence of ATP with 100 nM nicked DNA substrate (C:G 28mer) to determine the concentration of active LIG1 in solution. LIG1 WT and K845N were determined to be 97 ± 8% and 104 ± 7% active, respectively.

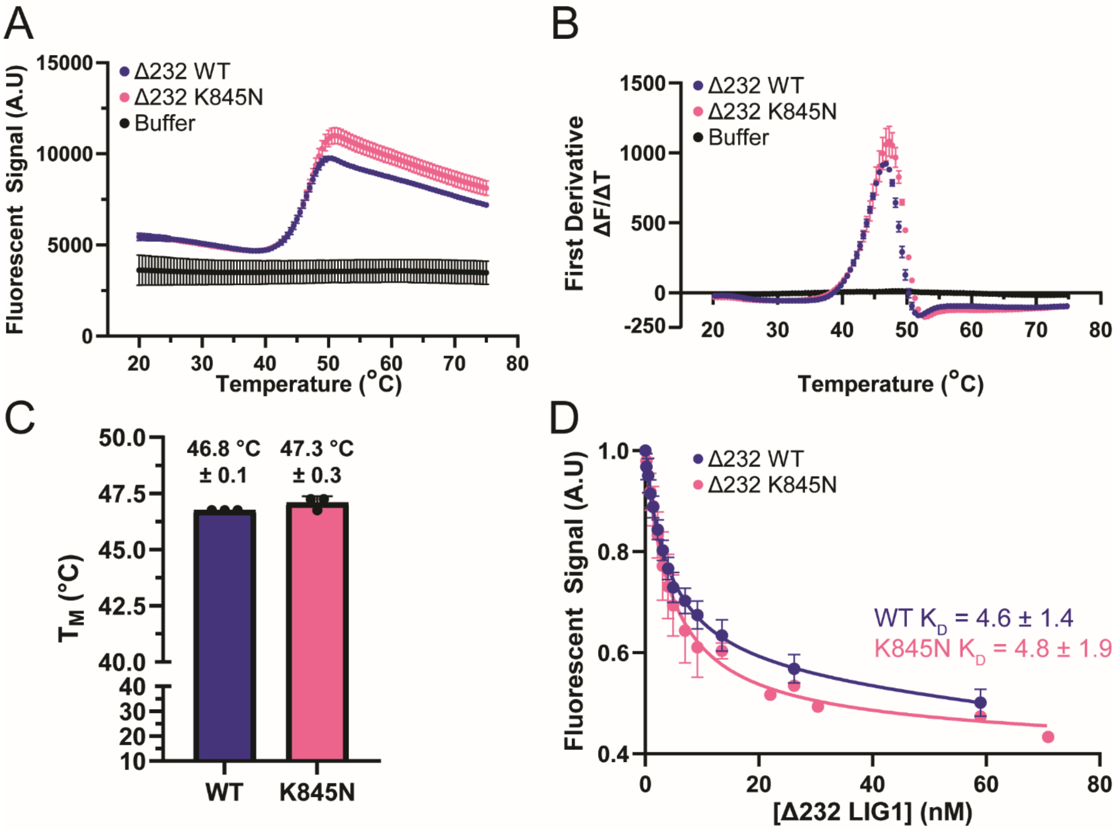

Figure S7. Comparison of LIG1 stability and DNA binding affinity for LIG1 K845N and WT. **(**A) Differential scanning fluorimetry was used to determine the melting temperature of Δ232 WT and K845N LIG1. 1 µM enzyme was mixed with SYPRO Orange dye (5X final concentration; Sigma). The experiment was carried out in 25 mM MOPS pH 7.5, 150 mM NaCl, 1 mM DTT and 2 mM EDTA. Melting data was collected using the CFX Opus 384 Real-Time PCR system with fluorescence data recorded every 0.5 °C/min (excitation 492 nm, emission 610 nm). (B) T_M values were calculated as the maximal value of the first derivative of the melting curve data. (C) Reported values represent the mean ± SD from two independent experiments, each with three technical replicates. (D) DNA binding was measured using a previously reported binding assay (4). Briefly, LIG1 variants were incubated in the presence of 0.5 nM fluorescein-deoxythymidine base (TFAM) nicked 44mer DNA substrate. The substrate is labeled with at the 3′-OH of the nick, which undergoes fluorescent quenching upon enzyme binding. Experiments were performed in 50 mM

MOPS pH 7.5, ionic strength adjusted to 150 mM with NaCl, 0.1 mg/mL BSA, 1 mM DTT, 1 mM EDTA. Data were fit to a one-site binding hyperbola to determine dissociation constants (K_D). Reported values represent the mean ± SD from three independent experiments.

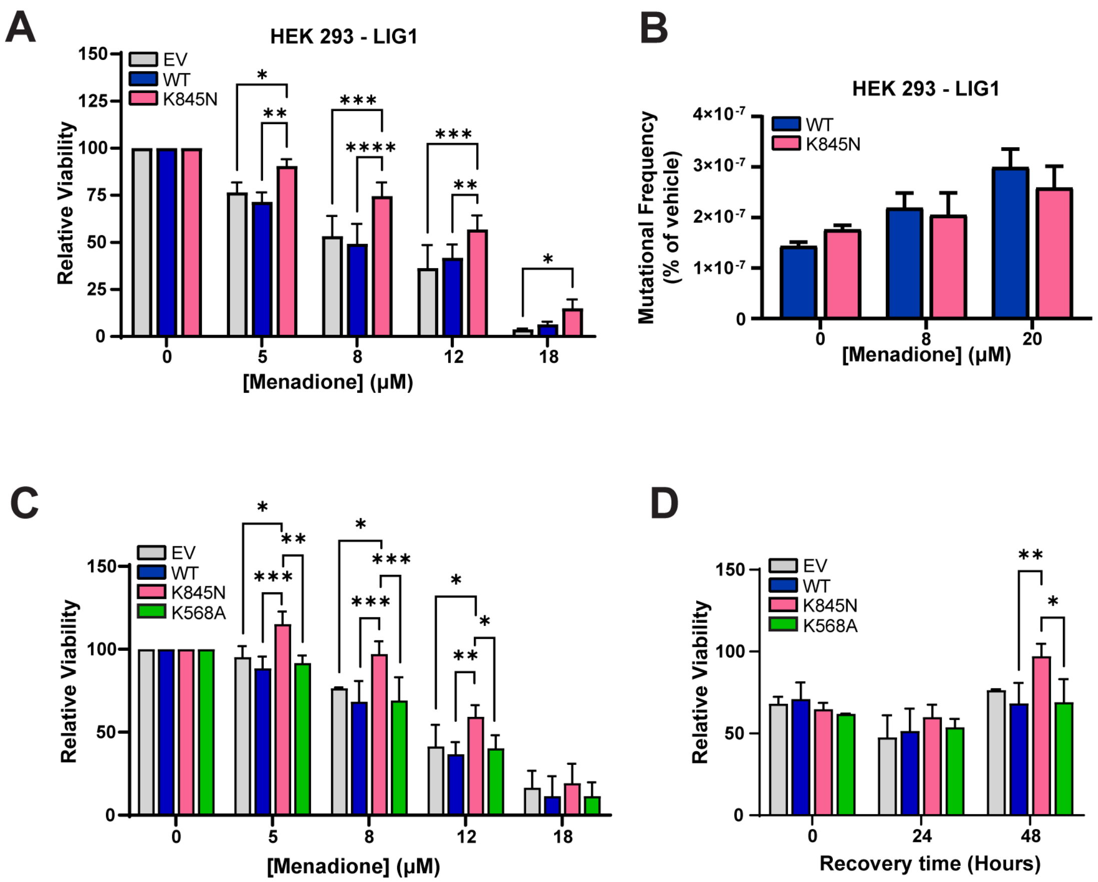

Figure S8. Analysis of oxidative stress response in HEK 293 EV, WT, K845N and K568A cells. (A) HEK 293 cells (EV, WT or K845N) were treated with menadione for 4 hours at the indicated concentrations. Cells were washed in fresh medium and incubated for 48 hours. Cellular viability was measured by CTG. (B) HEK 293 cells (WT or K845N) were treated with menadione for 4 hours at the indicated concentrations and washed in fresh medium. After 48 hours of incubation, cells were harvested for DNA extraction and mutation frequencies were determined by duplex sequencing. Bar graph shows mean mutation frequency (MF) of 2 biological replicates ± standard deviation. (C) HEK 293 cells (EV, WT, K845N or K568A) were treated with menadione for 4 hours at the indicated concentrations. Cells were washed in fresh medium and incubated for 48 hours. Cellular viability was measured by CTG. (D) HEK 293 cells (EV, WT, K845N or K568A) were treated with 8 μM menadione for 4 hours. Cells were washed in fresh medium and further incubated for the indicated times. All bar graph except (B) show mean percent viability relative to

baseline (0% menadione) of 3 biological replicates ± standard deviation. Statistical significance was determined from 2-way ANOVA with Tukey's multiple comparison test (****$p < 0.0001$, **$p < 0.01$, *$p < 0.05$).

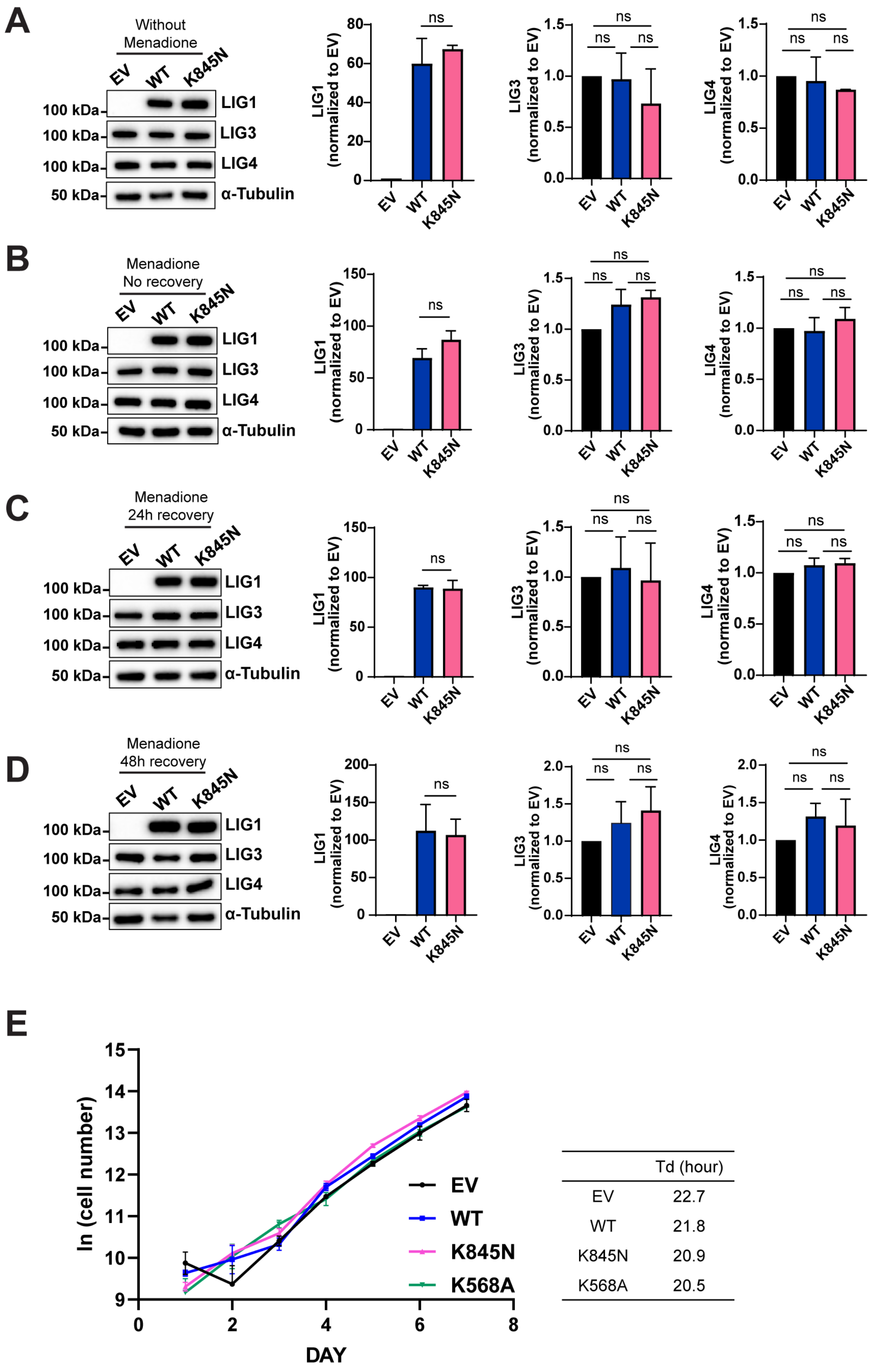

	Td (hour)
EV	22.7
WT	21.8
K845N	20.9
K568A	20.5

Figure S9. Analysis of DNA ligase expression and proliferation in HEK 293 EV, WT, and K845N cells. (A) Immunoblot analysis of LIG1, LIG3, and LIG4 in HEK 293 EV, WT, and K845N cells under basal (untreated) conditions. (B) Cells treated with menadione without recovery. (C) Cells treated with menadione followed by 24 h recovery. (D) Cells treated with menadione followed by 48 h recovery. Protein levels were quantified by normalizing the intensity of each ligase (LIG1, LIG3, LIG4) to α-tubulin, followed by normalization to EV controls. Statistical analysis was performed using one-way ANOVA with Tukey's multiple comparisons test. No significant differences in LIG1, LIG3, or LIG4 expression were observed among EV, WT, and K845N cells under any condition. (E) HEK 293 cells expressing empty vector (EV), WT LIG1, K845N LIG1 or K568A LIG1 were cultured for 7 days, and cell numbers were measured daily. Growth rates were calculated using the linear portion of the growth curve (days 3–7). The table shows the doubling time (Td) for each cell line derived from linear regression analysis. Linear regression revealed no significant differences in proliferation rate among the four cell lines ($p = 0.268$), indicating that overexpression of WT, K845N or K568A LIG1 does not alter baseline HEK 293 cell growth.

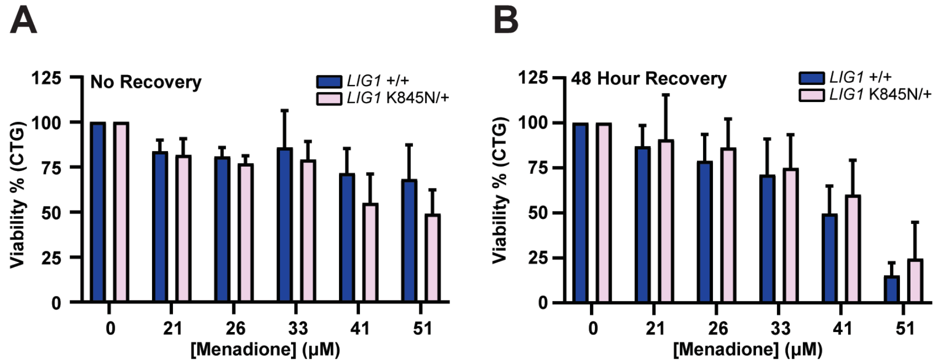

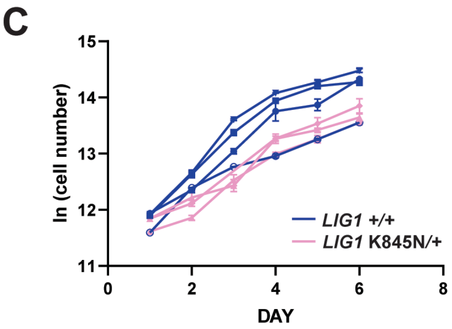

	HTT		Td (hour)
	CAG1	CAG2	
LIG1+/+	42	15	26.2
	41	17	39.0
	43	17	24.4
	47	17	22.6
LIG1 K845N/+	46	17	33.2
	46	17	35.7
	46	17	32.6

Figure S10. Comparison of cytotoxicity of HD LCLs expressing LIG1 WT or K845N under menadione-induced stress conditions. (A-B) HD LCLs homozygous for the *LIG1* rs145821638 (GRCh38 - Chr19:48117686) reference C-allele (*LIG1*+/+) or heterozygous for the 19AM3 modifier variant rs145821638 A-allele (*LIG1*K845N/+) (5) were treated with menadione for 4 hours at the indicated concentrations (see Fig.3 for details of LCLs). (A) Cell viability was measured immediately with no recovery time. (B) Cells were washed in fresh medium and incubated for 48 hours. Cellular viability was measured by CTG from the mean value of three technical replicates. Bar graph shows mean percent viability relative to baseline (0% menadione) ± standard deviation (n = 4 *LIG1* +/+ independent LCLs, n = 3 independent *LIG1* K845N/+ LCLs). Statistical significance indicated in figure was determined by 2-way ANOVA with Tukey's multiple comparison test. (C) HD LCLs were analyzed for proliferation rate. Cells were cultured for 6 days, and cell number was measured daily. For estimation of proliferation rate, growth rates were calculated using the linear portion of the growth curve (days 1–4) and doubling times (Td) for each LCL were calculated and summarized in the table. Due to variability within each group, a mixed-effects model was used to compare proliferation rates between the *LIG1* +/+ and *LIG1* K845N/+. No significant difference was detected ($p = 0.249$), indicating that LCLs carrying the K845N/+ do not exhibit altered proliferation relative to *LIG1* +/+.

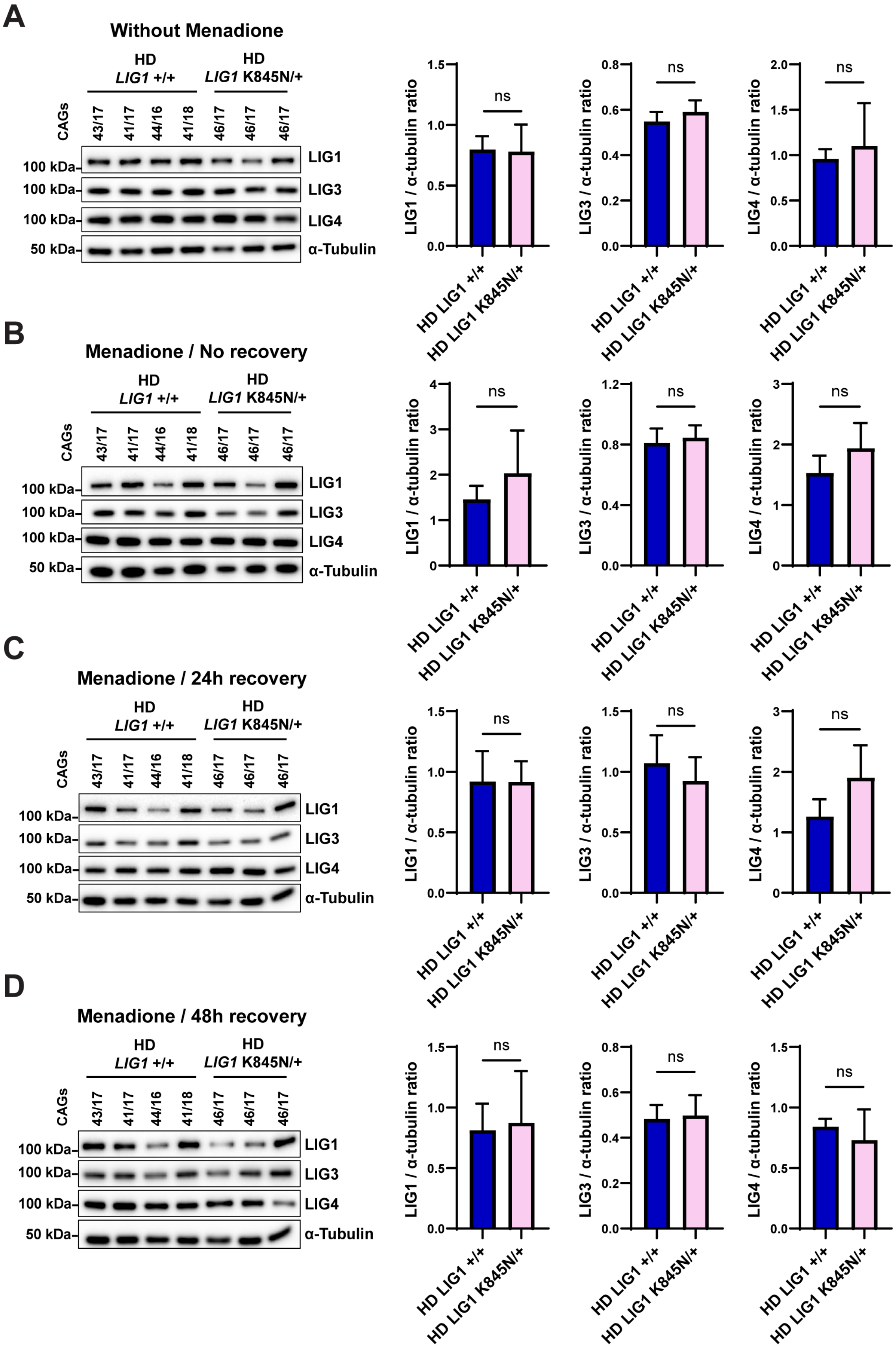
A
Without Menadione
B
Menadione / No recovery
C
Menadione / 24h recovery
D
Menadione / 48h recovery
HD
LIG1 +/+
HD
LIG1 K845N/+
CAGs
43/17
41/17
44/16
41/18
46/17
46/17
46/17
100 kDa
100 kDa
100 kDa
50 kDa
LIG1
LIG3
LIG4
α-Tubulin
LIG1 / α-tubulin ratio
LIG3 / α-tubulin ratio
LIG4 / α-tubulin ratio
ns
HD LIG1 +/+
HD LIG1 K845N/+

Figure S11. Expression analysis of LIG1, LIG3 and LIG4 in LCLs. HD LCLs were analyzed under four conditions. (A) untreated (basal), (B) 4 h menadione treatment with no recovery, (C) 4 h menadione treatment followed by 24 h recovery, and (D) 4 h menadione treatment followed by 48 h recovery. Protein levels were assessed by western blot, and band intensities were quantified by normalizing each target protein (LIG1, LIG3, LIG4) to α-tubulin. Statistical comparisons between the *LIG1* +/+ (n = 4) and *LIG1* K845N/+ (n = 3) groups were performed using unpaired t-tests with Welch's correction. Across all treatment conditions, no significant differences were observed in the expression of LIG1, LIG3, or LIG4 between the two groups.

Lig1 genotype	*Htt* genotype	Males	Females	Total
K834N/K843N	Q111/+	11	6	17
	+/+	7	16	23
	Combined	18	22	40
K834N/+	Q111/+	28	27	55
	+/+	23	25	48
	Combined	51	52	103
+/+	Q111/+	5	16	21
	+/+	12	14	26
	Combined	17	30	47

Test	Chi2, df	P val
Males, Observed vs. Expected, Combined *Htt* genotypes	1.716, 2	0.4240
Females, Observed vs. Expected, Combined *Htt* genotypes	0.6190, 2	0.7338
Total, Observed vs. Expected,, Combined *Htt* genotypes	1.058, 2	0.5891
Total, *Htt*Q111/+ vs. *Htt*+/+	1.824, 2	0.4017

Figure S12. Expected Mendelian ratios in transmission of the *Lig1*^{K843N} allele. Top: Number of pups (genotyped at weaning) in *Htt*$^{Q111/+}$ *Lig1*$^{K843N/+}$ x *Htt*$^{+/+}$ *Lig1*$^{K843N/+}$ crosses. Bottom: Chi square tests show lack of deviation from expected Mendelian ratios and no difference in Mendelian ratios between *Htt*$^{+/+}$ and *Htt*$^{Q111/+}$ backgrounds.

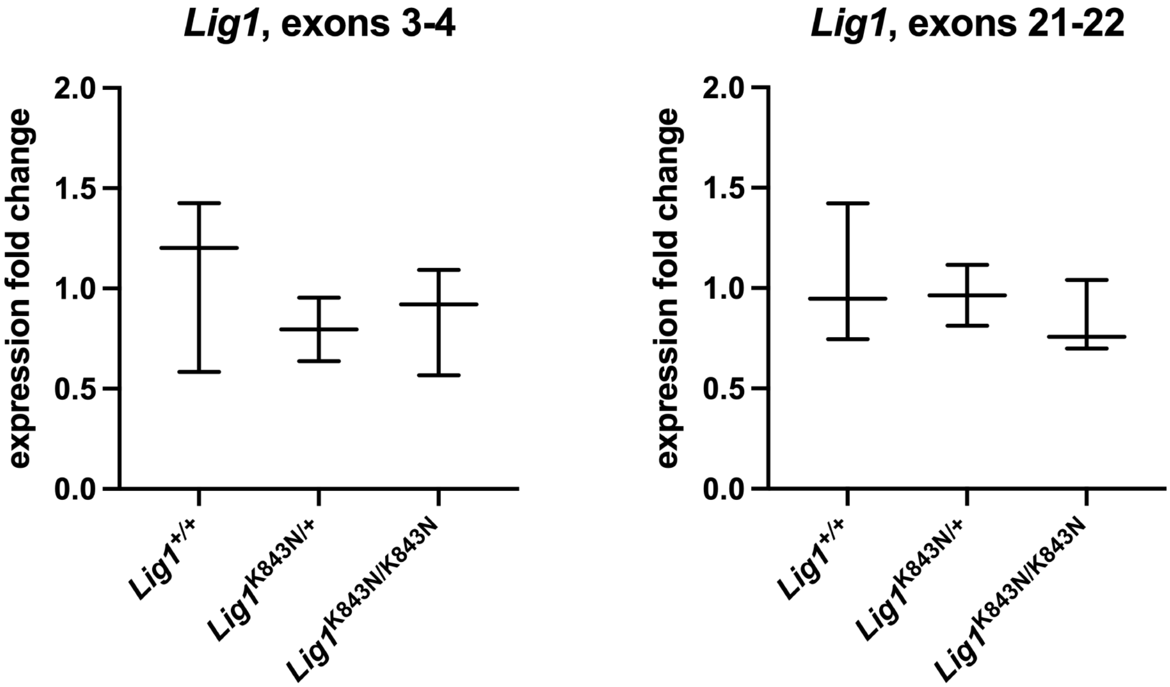

Figure S13. Impact of *Lig1*^{K843N} variant on *Lig1* expression. Quantitative PCRs using TaqMan assays of *Lig1* mRNA (exons 21-22 and exons 3-4) in mouse liver. Relative expression values (DC_t) of *Lig1* for each mouse were calculated using the geometric mean of the C_t values of three housekeeping genes *Ppia*, *Actb* and *Gusb*. Expression fold change (2^{-DDCt}) of *Lig1* for each mouse was calculated relative to the geomean of relative expression values (DC_t) of the control group (*Lig1*$^{+/+}$) mice. One way ANOVA with Tukey's multiple comparison test did not show significant differences between genotypes. *Lig1*$^{+/+}$ N=3, *Lig1*$^{K843N/+}$ N=2, *Lig1*$^{K843N/K843N}$ N=3. Two technical replicates were performed for each mouse tissue, which were averaged.

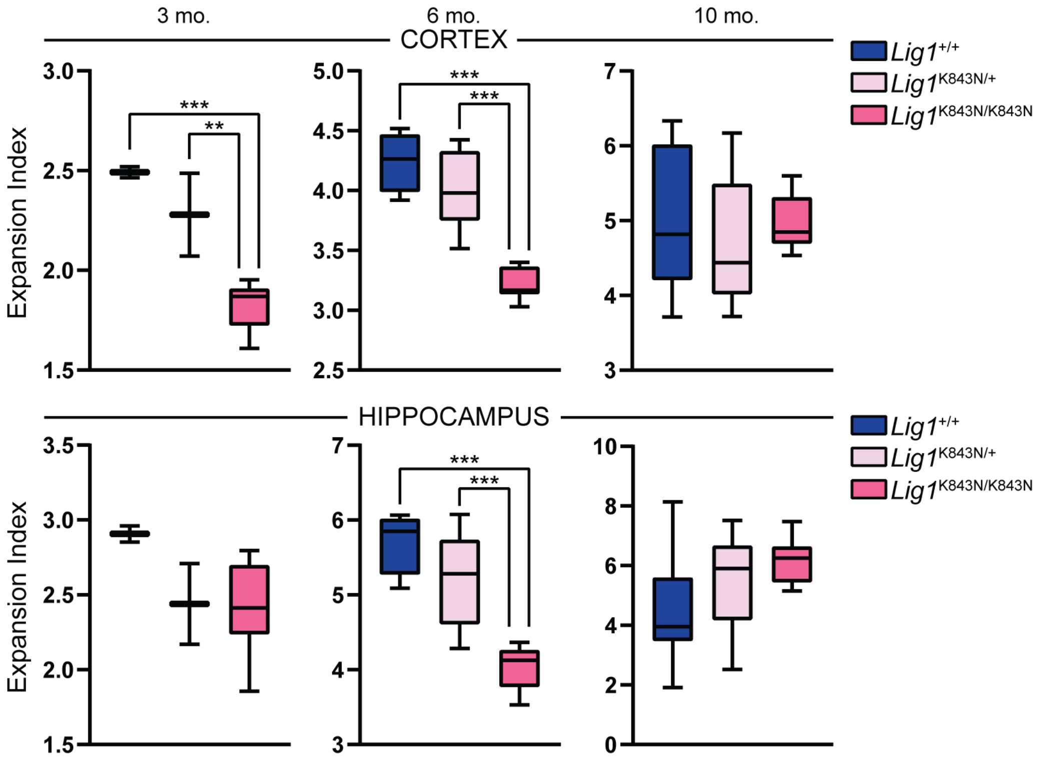

Figure S14. Impact of *Lig1*^{K843N} variant on somatic expansion in cortex and hippocampus. Somatic CAG expansion indices (Min to Max box-whisker plots) from cortex and hippocampus of *Htt*$^{Q111/+}$ mice with different *Lig1* genotypes. 3 mo: *Lig1*$^{+/+}$ N=2, *Lig1*$^{K843N/+}$ N=2, *Lig1*$^{K843N/K843N}$ N=8. 6 mo: *Lig1*$^{+/+}$ N=4, *Lig1*$^{K843N/+}$ N=9, *Lig1*$^{K843N/K843N}$ N=7. 10 mo: Lig1+/+ N=9, *Lig1*$^{K843N/+}$ N=9, *Lig1*$^{K843N/K843N}$ N=10. **p < 0.01; ***p < 0.001, ****p < 0.0001 (One way ANOVA, comparing all genotypes for each age and tissue, with Tukey's multiple comparison test).

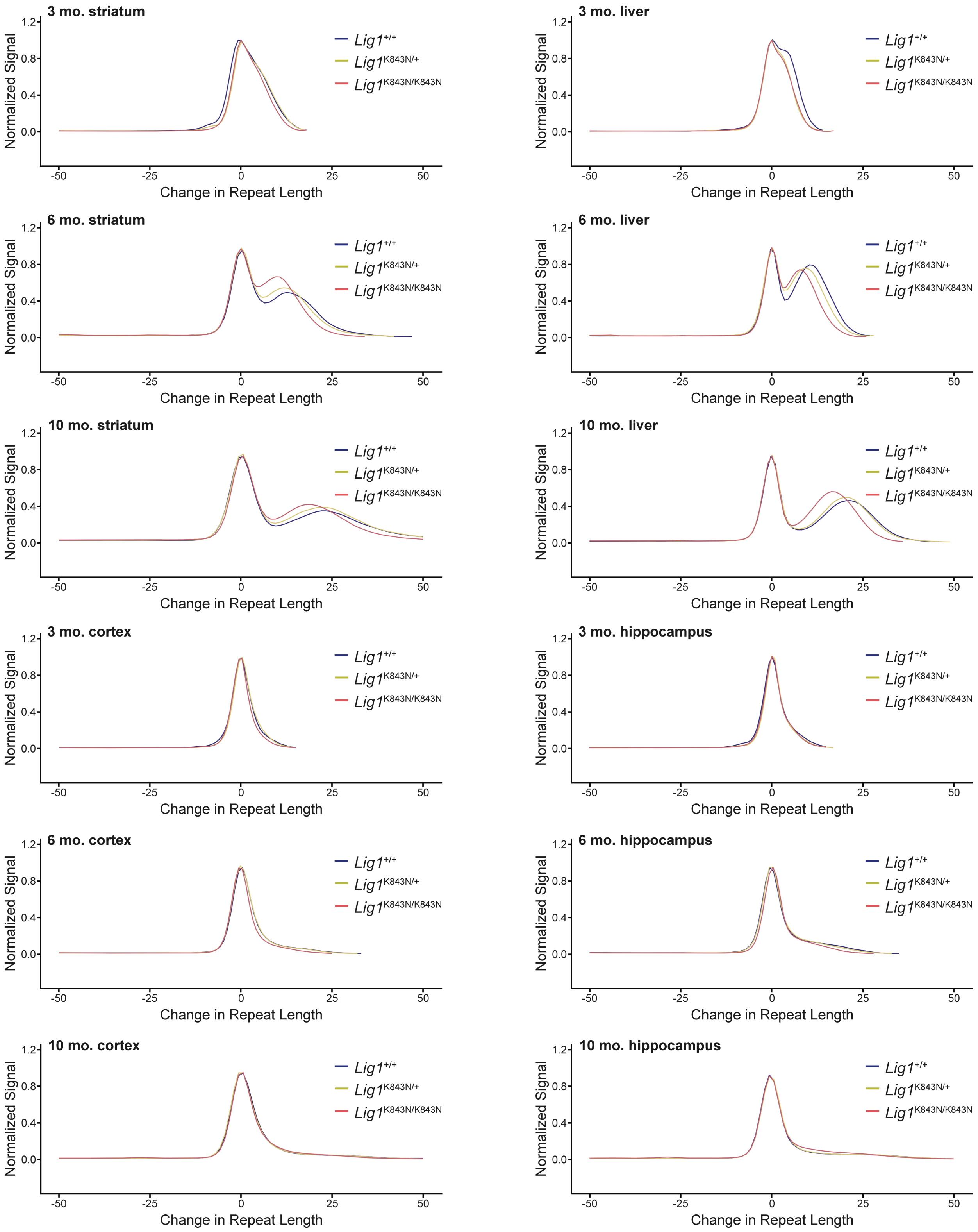
3 mo. striatum
3 mo. liver
6 mo. striatum
6 mo. liver
10 mo. striatum
10 mo. liver
3 mo. cortex
3 mo. hippocampus
6 mo. cortex
6 mo. hippocampus
10 mo. cortex
10 mo. hippocampus
Normalized Signal
1.2
0.8
0.4
0.0
Change in Repeat Length
-50
-25
0
25
50
Lig1+/+
Lig1K843N/+
Lig1K843N/K843N

Figure S15. Repeat length distributions. Peak height and repeat length data were extracted from ABI 3730 fsa files using Traceshiny software (Traceshiny.mgh.harvard.edu). For each age-tissue combination, an average trace was generated across all mice of one genotype, normalizing the y-axis heights to 1 and adjusting the x-axis such that the modal allele is zero, in order to compare between genotypes. 3 mo: *Lig1*$^{+/+}$ N=2, *Lig1*$^{K843N/+}$ N=2, *Lig1*$^{K843N/K843N}$ N=8. 6 mo: *Lig1*$^{+/+}$ N=4, *Lig1*$^{K843N/+}$ N=9, *Lig1*$^{K843N/K843N}$ N=7. 10 mo: Lig1+/+ N=9, *Lig1*$^{K843N/+}$ N=9, *Lig1*$^{K843N/K843N}$ N=10. Although distributions may differ subtly between genotypes, quantification of an expansion index that includes a weighting factor for the distance from the modal allele (*i.e.* longer expansions have a greater weight) across the entire range of repeats can distinguish genotypes as shown in Figure 4. Blue: *Lig1*$^{+/+}$; Yellow: *Lig1*$^{K843N/+}$; Pink: *Lig1*$^{K843N/K843N}$

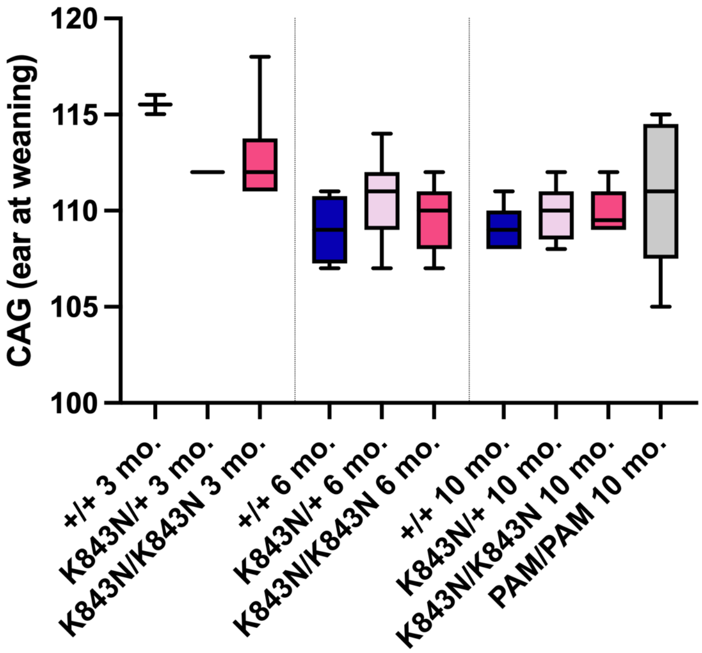

Figure S16. Inherited CAG lengths in mice cohorts. CAG lengths (Min to Max box-whisker plots) measured in ear tissue at weaning in 3-month, 6-month and 10-month cohorts. 3 mo: *Lig1*$^{+/+}$ N=2, *Lig1*$^{K843N/+}$ N=2, *Lig1*$^{K843N/K843N}$ N=8. 6 mo: *Lig1*$^{+/+}$ N=4, *Lig1*$^{K843N/+}$ N=9, *Lig1*$^{K843N/K843N}$ N=7. 10 mo: Lig1+/+ N=9, *Lig1*$^{K843N/+}$ N=9, *Lig1*$^{K843N/K843N}$ N=10, *Lig1*$^{PAM/PAM}$ N=5. *Lig1*$^{PAM/PAM}$ mice are homozygous for the silent C>T PAM mutation but are wild-type for the K843N variant. One way ANOVA, comparing all genotypes for each age, with Tukey's multiple comparison test, showed no significant differences in inherited CAG lengths between mice of different *Lig1* genotypes.

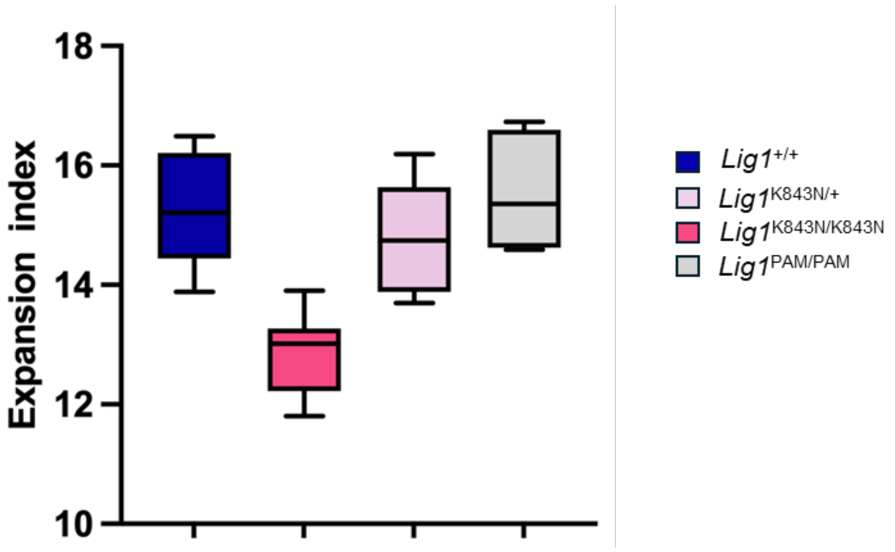

Figure S17. Silent PAM mutation alone does not alter CAG expansion. Somatic CAG expansion indices (Min to Max box-whisker plots) from liver of 10-month *Htt*$^{Q111/+}$ mice with different *Lig1* genotypes. *Lig1*$^{+/+}$ N=9, *Lig1*$^{K843N/+}$ N=9, *Lig1*$^{K843N/K843N}$ N=10, *Lig1*$^{PAM/PAM}$ N=5. One way ANOVA with Tukey's multiple comparison test comparing all genotypes, showed no significant difference between *Lig1*$^{+/+}$ and *Lig1*$^{PAM/PAM}$ expansion indices (p=0.9552), and significantly lower expansion indices in *Lig1*$^{K843N/K843N}$ mice compared to *Lig1*$^{PAM/PAM}$ mice (p < 0.0001). *Lig1*$^{PAM/PAM}$ mice are homozygous for the silent C>T PAM mutation but are wild-type for the K843N variant.

WT mouse striatum (Lee et al. 2020)

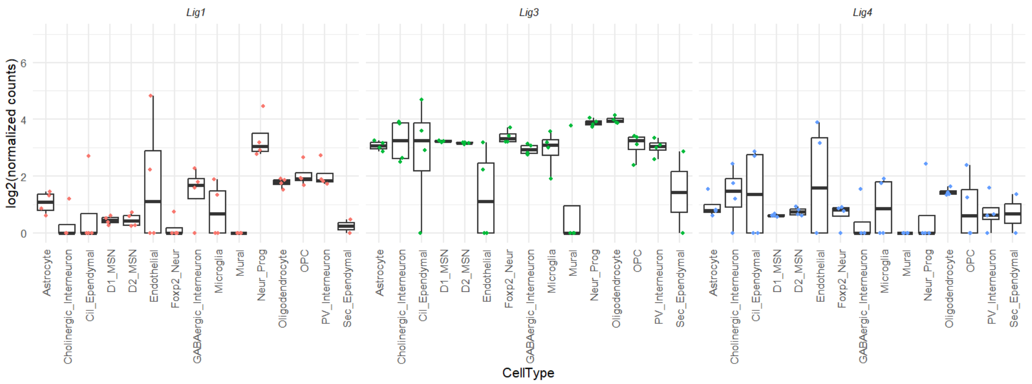

Control human striatum (Handsaker et al. 2025)

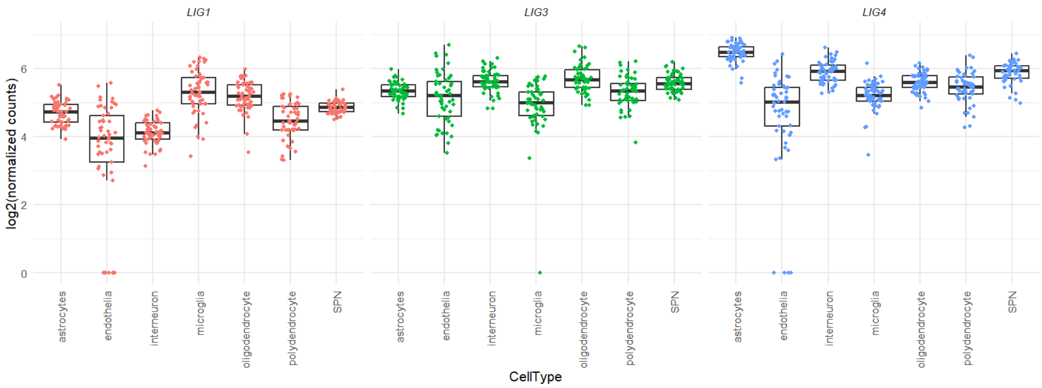

Figure S18. Expression of DNA ligase genes in the striatum. Single nuclear RNA sequencing (snRNA-seq) data from wild-type mice striata were obtained from Lee et al., Neuron 2020 Sep 9;107(5):891-908.e8, sourced from the NCBI GEO under accession #GSE152058. snRNA-seq data from human striatum were obtained from Handsaker et al., Cell 2025 Feb 6;188(3):623-639.e19, sourced from NeMO the Neuroscience Multi-omic Archive, under accession dat-ztfn3cc. In each case, normalized gene expression values were obtained from the pseudobulked count matrix using size-factor normalization (*counts(dds, normalized = TRUE)*). For downstream visualization, normalized counts for the selected genes were extracted and transformed as *log2(count + 1)* to stabilize variance and avoid undefined values at zero. Cil_Ependymal: ciliated ependymal cell; dSPN: direct pathway spiny projection neuron; iSPN: indirect pathway spiny projection neuron; Foxp2_Neur: Foxp2/Olfm3 neuron; Neur_Prog: Neuronal_Progenitor cells; OPC: oligodendrocyte progenitor cell; PV_Interneuron: parvalbumin interneuron; Sec_Ependymal: secretory ependymal cell.

Table S1. Oligonucleotides for ligation assays

Oligo Name	Sequence (5’ – 3’)
Up18OH-A	CAT GGG CGG CAT GAA CCA
Up18OH-G	CAT GGG CGG CAT GAA CCG
Up18OH-C	CAT GGG CGG CAT GAA CCC
Up18OH-T	CAT GGG CGG CAT GAA CCT
Up18OH-^{oxo}G	CAT GGG CGG CAT GAA CC^{oxo}G
DownP16-FAM	PO4-GAG GCC CAT CCT CAC C-FAM
Temp34-T	GGT GAG GAT GGG CCT CTG GTT CAT GCC GCC CAT G
Temp34-C	GGT GAG GAT GGG CCT CCG GTT CAT GCC GCC CAT G
Temp34-A	GGT GAG GAT GGG CCT CAG GTT CAT GCC GCC CAT G
Temp34-G	GGT GAG GAT GGG CCT CGG GTT CAT GCC GCC CAT G
Up13OH-C	GTGCTGATGCGTC
UP13OH-^{oxo}G	GTGCTGATGCGT^{oxo}G
DownP15-FAM	PO4-GTCGGACTGATTCGG-FAM
Temp28-G	CCGAATCAGTCCGACGACGCATCAGCAC
Temp28-A	CCGAATCAGTCCGACAACGCATCAGCAC

The indicated Up18OH, P16-FAM, and Temp34 strand (Caglayan 2017) were annealed for ligation assays with the full-length LIG1. The indicated Up13OH, DownP15-FAM, and Temp28 strand (Tumbale 2019) were annealed for assays with Δ232 LIG1. Annealing ratios were 1:1.5:2 with respect to the phosphate, template, and 3′OH strands.

Table S2. Steady-state kinetic parameters for Δ232 LIG1

	C:G Nicked DNA		
	WT	K845N	Ratio K845N to WT
k_{cat} (s^{-1})	0.52 ± 0.01	0.20 ± 0.01	0.38
K_M (nM)	41.6 ± 5.9	80.7 ± 13.8	1.9
k_{cat}/K_M($M^{-1}s^{-1}$)	12.5 ± 1.8 x10^6	2.5 ± 0.4 x10^6	0.20
Abortive Ligation	0.01 ± 0.00	0.028 ± 0.003	

	8-oxoG:A Nicked DNA		
	WT	K845N	Ratio K845N to WT
k_{cat} (s^{-1})	0.08 ± 0.01	3x10^{-3} ± 5x10^{-4}	0.038
K_M (nM)	31.7 ± 5.9	73.3 ± 16.5	2.3
k_{cat}/K_M($M^{-1}s^{-1}$)	2.4 ± 0.5 x10^6	0.04 ± 0.01 x10^6	0.017
Abortive Ligation	0.51 ± 0.03	0.93 ± 0.01	

	Fidelity Measurements		
	WT	K845N	Ratio K845N to WT
Discrimination against 8-oxoG:A	5.1 ± 1.3	60.1 ± 19.6	12

These data are from experiments described in Fig. 2. Reactions were performed in standard reaction buffer at 1.2 mM $MgCl_2$ (1 mM Free Mg^{2+}) and 0.2 mM ATP. Values are the average ± SD (n ≥ 3).

References

1. J. M. Pascal, P. J. O'Brien, A. E. Tomkinson, T. Ellenberger, Human DNA ligase I completely encircles and partially unwinds nicked DNA. *Nature* **432**, 473–478 (2004).
2. F. W. Studier, Protein production by auto-induction in high density shaking cultures. *Protein Expr Purif* **41**, 207–234 (2005).
3. P. P. Tumbale *et al.*, Two-tiered enforcement of high-fidelity DNA ligation. *Nat Commun* **10**, 5431 (2019).
4. T. J. Jurkiw *et al.*, LIG1 syndrome mutations remodel a cooperative network of ligand binding interactions to compromise ligation efficiency. *Nucleic Acids Res* **49**, 1619–1630 (2021).
5. GeM-HD, Genetic modifiers of somatic expansion and clinical phenotypes in Huntington's disease highlight shared and tissue-specific effects. *Nat Genet* **57**, 1426–1436 (2025).
